# Optimal exercise parameters of Baduanjin for balance in older adults: a systematic review and meta-analysis

**DOI:** 10.3389/fpubh.2025.1541170

**Published:** 2025-03-19

**Authors:** Yikun Yang, Enjing Li, Zixin Gong, Mauri Tualaulelei, Zhiwei Zhao, Zhiyan Zhang

**Affiliations:** ^1^School of Physical Education and Sport, Central China Normal University, Wuhan, China; ^2^School of Wushu, Wuhan Sports University, Wuhan, China; ^3^School of Electronic Engineering, Xi'an University of Posts and Telecommunications, Xian, China

**Keywords:** dosages, balance, Baduanjin, Qigong, traditional exercise

## Abstract

**Purpose:**

Baduanjin represents an affordable and secure method of exercising both the mind and body, and has been observed to enhance balance in older adults. Nevertheless, the existing research on the impact of Baduanjin on various balance functions is still insufficient, and the optimal dosage parameters for performing Baduanjin exercises have not been studied.

**Methods:**

To conduct a systematic review and meta-analysis, five English databases and three Chinese databases were performed. Relevant studies were searched by GOOGLE SCHOLAR, Web of Science, Cochrane, Embase, Pubmed, CNKI, SinoMed, and WangfangMed using “Baduanjin” and “balance.” Subgroup analyses were conducted to investigate the influence of different exercise parameters on the observed outcomes. Meta-regression was employed to identify potential moderating factors. The Physical Therapy Evidence Database (PEDro) scale was used for quality assessment.

**Results:**

A total of 40 studies were included in the analysis, while the majority of studies report promising outcomes, the overall quality of these studies is relatively low. The results of the meta-analysis showed Baduanjin significantly enhanced static balance (SMD = 0.87, 95%CI: 0.69–1.05), dynamic balance (SMD = 0.85, 95%CI: 0.50–1.19), proactive balance (SMD = −1.00, 95%CI: −1.33–−0.67) and balance test battery (SMD = 1.04, 95%CI: 0.81–1.28) in older adults. Additionally, the findings indicated a notable reduction in the risk of falls (SMD = −2.19, 95%CI: −3.35–−1.04) and an improvement in fall efficacy (SMD: 0.57, 95%CI: 0.36–0.78).

**Conclusion:**

Baduanjin has been demonstrated to improve balance and reduce the risk of falls in older adults. Studies have found that significant gains begin to be achieved with a minimum of 12 weeks of practice and that 30–49 min of practice, 5–7 times per week, works best for developing different balances. However, most of the included studies were limited by a lack of blinding and follow-up visits, and there was an insufficient number of short-term or long-term studies to establish complete optimal parameters robustly.

**Systematic review registration:**

https://www.crd.york.ac.uk/prospero/display_record.php?RecordID=548345, identifier: CRD42024548345.

## 1 Introduction

Falls represent a significant health concern for middle-aged and older adults, leading to fractures, bruises, and other injuries, and in severe cases, even death (1, 2). According to the World Health Organization (WHO), approximately 684,000 deaths globally are attributed to falls each year, with the majority occurring in individuals over 60 (3). Falls have become the second leading cause of accidental death globally, with the likelihood of experiencing a fall and subsequent hospitalization increasing with age (4). Annually, there are 37.3 million serious falls requiring medical attention, with older adults comprising the largest demographic among fall victims (3). Balance disorders are recognized as key internal factors contributing to falls in older adults (5). Those with poor balance are at a higher risk of future falls (6), and balance tests are often employed to predict fall occurrences (7). Balance training has emerged as an effective strategy for fall prevention, with both traditional exercises and advanced technologies like virtual reality offering diverse options for middle-aged and older adults (8). Incorporating physical activity modalities that enhance balance—like core strength training or Pilates—has proven effective in improving balance and reducing fall risk (9).

In the 5,000-year history of the Chinese nation, a diverse array of traditional physical activities has emerged, with as many as 977 documented forms (10). Among these, substantial evidence has highlighted the health benefits of practices such as Tai Chi, Qigong, and Yangko dance, particularly for older adults (11). These benefits include reduced risk of influenza (12), improved quality of life for cancer patients (13), and enhanced quality of life for individuals with essential hypertension (14). Tai Chi, recognized as a renowned method for improving balance, has been extensively studied since the early 2000s (15, 16), and encouraging evidence supports its recommendation by the Centers for Disease Control and Prevention (CDC) as a fall prevention intervention for the older adults (16, 17).

Similar to the movement form of Taijiquan, Fitness Qigong, despite having different connotations, has also attracted the attention of colleagues, who discuss its benefits for older adults balance, includes Wuqinxi, Liuzijue, Mawangdui Daoyin, etc. (18). Baduanjin is one of the most renowned forms of Qigong, It comprises eight movements that emphasize weight shifting, muscle stretching, breathing exercises, and meditative training. Characterized by its simplicity and ease of learning, Baduanjin (BDJ) is highly implementable and remarkably safe, rendering it exceedingly popular among the older population.

Recent reviews indicate that BDJ can significantly improve balance and prevent falls in individuals over 65 years old (19). Additionally, it is recognized as an effective rehabilitation modality for enhancing balance and quality of life in stroke and Parkinson's patients (20, 21). BDJ also promotes lower extremity strength, flexibility, and overall balance in older adults (22, 23). Notably, BDJ boasts a strong safety profile with a low likelihood of serious adverse events (24), making it a favorable choice for older adults, particularly those in a weakened state.

While numerous randomized controlled trials have highlighted the positive effects of BDJ on balance in older adults, there is limited evidence addressing its impact on balance specifically broad evidence synthesis. Furthermore, no studies have identified the optimal dosage of BDJ for balance improvement. Therefore, to promote the scientific practice of Baduanjin (BDJ) exercise among older adults and to inform nursing practices aimed at improving balance in this population, this study seeks to address the following questions: (1) To supplement existing evidence regarding balance enhancement and fall prevention in older adults through BDJ; (2) To analyze the moderators influencing the relationship between BDJ and balance; (3) To explore the dose-response relationship between BDJ and balance.

## 2 Methods

This study was planned following the Preferred reporting items for systematic review and meta-analysis (PRISMA) (25), and was registered in the center of the International Prospective Systematic Evaluation Registry database (PEOSPERO) (registration number: CRD42024548345). The specific research process developed was guided by the JBI Manual for Evidence Synthesis (26).

### 2.1 Eligibility criteria

The study included older adults participating in BDJ exercise without strict limitations regarding race, gender, or health status. For the definition of “older adults,” we referred to other studies, considering mean aged 60 years or older (27), with no stringent health status criteria. However, we excluded individuals with disability or special situation (such as visual impairments, vestibular injuries, etc.). In terms of intervention modalities, we included studies that specifically utilized BDJ as an intervention. Studies incorporating sit-to-stand BDJ, mixed intervention programs, or failing to highlight the independent benefits of BDJ were excluded.

There were no strict restrictions on the control group, which included various interventions such as rehabilitation, routine care, health education, and walking. However, controls with strong functional directionality, such as other forms of Qigong, resistance training, and balance training, were excluded. The primary indicators included balance-related outcomes, categorized into five areas: (I) Static Balance (e.g., Eye Closed One Leg Standing Balance, One Leg Stand Test); (II) Dynamic Balance (e.g., Five Times Sit to Stand Test, Six-Minute Walk Test); (III) Proactive Balance; (IV) Passive Balance; and (V) Balance Test Batteries (including comprehensive tests like the Berg Balance Scale). Secondary indicators included fall rates, the number of falls, assessment results (e.g., Morse Fall Assessment Scale), and safety. Only controlled trials published in English or Chinese were included in this study. The original authors were to be contacted via email for any incorrect or incomplete data; if no response was received before data analysis commenced, the study was excluded. There were no restrictions regarding randomization, allocation concealment, blinding methods, or publication date.

### 2.2 Information sources

To ensure a comprehensive search, we utilized the portfolio of English databases outlined in the study by Bramer et al. (28), which included, at a minimum, Embase, MEDLINE (PubMed), Web of Science, and Google Scholar (top 200 items). Additionally, we supplemented our search with the Cochrane Library (Cochrane Database of Systematic Reviews and Cochrane Central Register of Controlled Trials). For Chinese databases, we included the China Knowledge Network, Chinese Biomedical Literature Database, and Wanfang Database. The search will remain open until May 1, 2024. To maximize the retrieval of all relevant studies, we will also review the reference lists of included studies and related systematic reviews once the initial search is complete.

### 2.3 Search strategy

The search terms are based on the PICO principle, and in the English databases, they include (“Old” OR “Aged” OR “older” OR “Senior”) AND (“Baduanjin” OR “Qigong” OR “Eight Section Brocades” OR “Traditional Chinese Exercise”) AND (“Balance” OR “Postural Balance” OR “Postural Stability” OR “Falling” etc.), and the search keywords in the Chinese database are slightly modified based on the English terms. The complete search strategies for different databases are provided in Appendix A.

### 2.4 Data selection and collection process

Once the two reviewers complete the search, the data will be imported into EndNote X9.1, with any data that cannot be imported automatically added manually. Following data collection, an automatic check for duplicates will be conducted, accompanied by a manual review. A third researcher will then review the results of this process. All data will be merged into a single dataset, with duplicate studies removed. Two independent reviewers will screen eligible articles based on pre-established criteria after reviewing the title, abstract, and full text, consulting a third reviewer in case of disagreements. Data extraction will be performed using standardized forms to capture basic information such as the title, first author, year of publication, study design, sample size, baseline characteristics, control groups, exit rate, follow-up details, outcomes, adverse events, and dosage information (duration, training frequency, intensity). In cases of data inconsistencies, a third reviewer will mediate until a unanimous agreement is reached. Any data errors will be reported to a third reviewer, who will organize an error check and exclude any literature with confirmed errors. For ambiguous or missing data, the third reviewer will contact the corresponding author via email for clarification, and if there is no response within 2 weeks, the study will be excluded.

### 2.5 Methodological quality assessment

This review utilized the PEDro scale to assess the methodological quality of primary studies (29). Two independent reviewers conducted the assessment, with a third reviewer facilitating discussions when disagreements arose. The PEDro scale comprises 11 items designed to evaluate the quality of studies in the Physical Therapy Evidence Database (PEDro), including one item for external validity, eight for internal validity, and two for statistical reporting. Researchers determined the quality of each study by summing the total scores, with interpretations as follows: scores <4 are considered “poor,” 4 to 5 are “fair,” 6 to 8 are “good,” and 9 to 10 are “excellent.” This approach has demonstrated reliability (30, 31).

### 2.6 Statistical analysis

This systematic review and meta-analysis employed a null hypothesis significance test by reporting Q. The degree of heterogeneity among the effect sizes of the studies was assessed using I^2^ and the heterogeneity statistic (H statistic). If a random effects model was utilized, an additional measure of absolute heterogeneity, τ^2^ (Tau squared), was reported. The I^2^ thresholds for relative heterogeneity were defined as follows: low (25%), moderate (50%), and high (75%) (32).

Considering the possible differences in magnitude of the different measures, this study combined the balance-related indicators by standardized mean deviation (SMD), collecting the sample sizes of the experimental and control groups, and the mean and the standard deviation for the calculation of the effect sizes. SMD was calculated for each study by SMD = (Mean1 − Mean2)/SDpooled, where the combined standard deviation was calculated using SDpooled = sqrt((n1 *SD1^2^ + n2 * SD2^2^)/(n1 + n2)). After calculating the weights for each study, SMDcombined = Σ(wi * SMDi)/Σwi was used for merging.

More conservative estimates will be made using a random effects model when heterogeneity is detected, and 95% confidence intervals for the combined effect sizes will be calculated. In contrast, a fixed effects model will be employed when heterogeneity is not present. All combined results are reported via forest plots. For studies where data merging was not feasible, narrative analysis was conducted after coding the data to engage in adding to the evidence. There may have been selective reporting of data in the included trials, with positive data being more likely to be published compared to negative data (33). If sufficient studies were available, publication bias was observed through funnel plots and the symmetry of the funnel plots was tested by Egger's test and Begg's test (34, 35).

This review used meta-regression to detect potential effects of level covariates on effect sizes. Covariates included exercise dose, control group, health status, and age. For those with significant heterogeneity, subgroup analyses were performed. Quantitative analyses were performed through the automated data analysis platform SPSSAU (version 24.0) (36), Revman 5.3 and SPSS 28.0.

## 3 Results

### 3.1 Literature search results

**Figure 1:**
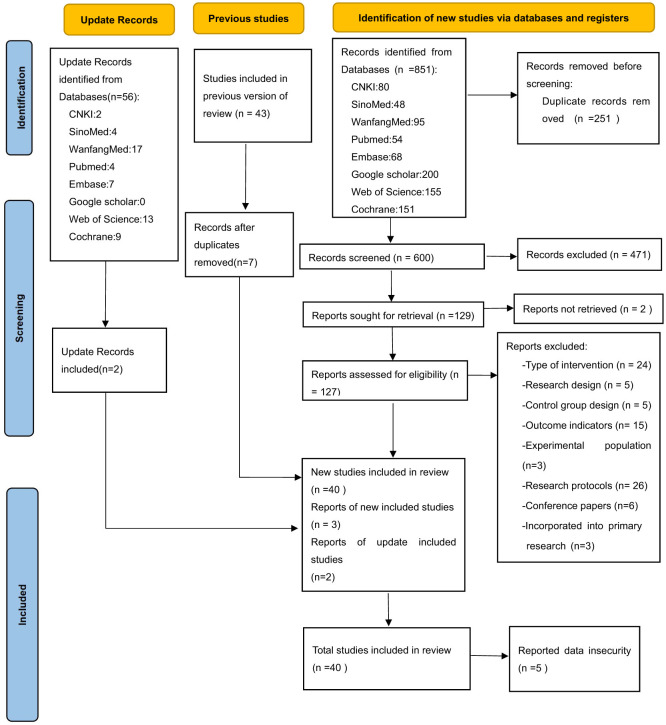
PRISMA 2020 flow diagram.

Figure 1 illustrates the process of literature inclusion, and total of 851 records were retrieved from 8 distinct databases. After two rounds of deduplication, 600 records remained. Upon reviewing the abstracts and titles, we incorporated 127 studies into our analysis. However, following a thorough examination of the complete articles, we excluded 87 studies. The primary reasons for exclusion were mismatched intervention methods, absence of relevant outcome indicators, research protocols, and conference proceedings. Additionally, three studies were incorporated by reviewing previous versions of the systematic review. During the data extraction phase, data security concerns were identified in five studies from the same institutions, reducing the final sample to 38 studies (37–74). The search was updated on January 22, 2025, resulting in 55 revised records. Following the screening process, 2 additional studies were included (75, 76). In the aggregate, a total of 40 studies were incorporated into the analysis in two successive phases.

### 3.2 Study characteristics

A detailed summary of the characteristics of these studies is provided in Appendix B. The mean age of participants ranged from 60 to 75 years across 35 studies (37, 39, 43–57, 59–64, 66–76), and from 75 to 85 years in 5 studies (40–42, 58, 65). The dataset comprised 30 journal articles (37, 38, 41, 43, 45, 46, 48, 50–54, 57, 59–75) and 10 theses (39, 40, 42, 44, 47, 49, 55, 56, 58, 76), with two studies were conducted in Spain (67) and Singapore (75), and the remainder in China. Participants were categorized by health status as either generally healthy or incompletely healthy. “Generally healthy” was defined as the absence of mobility or psychological disorders, normal cognitive function, basic balance ability, and no serious acute illnesses or diseases affecting balance. Of the total, 16 studies (37–39, 42, 46, 47, 49, 52, 55–57, 62, 67–69, 76) involved generally healthy participants, 23 studies (40, 41, 43, 44, 48, 50, 51, 53, 54, 58–61, 63–66, 70–75) included incompletely healthy participants, and one study (45) did not report the health status of participants.

In alignment with the definitions of balance ability indicators outlined in previous studies (77, 78), the outcome measures in this review encompass static balance indicators (SBT, including the single-leg standing test and related kinematic measures), dynamic balance indicators (DBT, including the 6-min walk test), proactive balance indicators (PBT, including the timed up and go test), the balance test batteries (BTB, including the mountain balance test), and passive balance. Among the studies included in the review, 22 reported on SBT (37–40, 42, 45, 49, 50, 52, 54–56, 62, 64, 67–72, 74), 16 on DBT (37–40, 45, 48–50, 53, 58, 63, 65, 66, 72, 75, 76), 20 on PBT (38, 40, 42, 43, 45, 49, 50, 52–54, 56, 58, 59, 64, 68–70, 72, 75, 76), and 21 on BTB (38, 39, 41, 43, 44, 51, 52, 54–56, 58–61, 64, 66, 68–70, 73, 76). Additionally, 14 studies included fall-related indicators (40, 43, 46, 47, 49, 50, 54, 57, 59, 64, 66, 68, 72, 75).

Table 1 summarizes the primary outcomes, participant characteristics, and information on drug and alcohol consumption of the included studies. While the majority of studies reported positive outcomes, the high-quality study by Tou et al. (75) found no significant differences in balance measures or fall efficacy between the intervention and control groups following a 4-month intervention. Discrepancies in findings have also emerged regarding static balance assessments utilizing force plates or plantar pressure sensors, with some studies reporting less favorable outcomes. Nevertheless, the majority of studies demonstrated significant improvements in test items such as the Timed Up and Go (TUG) test, 6-Min Walk Test (6WMT), and Five Times Sit to Stand Test (FTSST), etc. The primary results included in the study are (1) Balance test batteries, (2) Proactive balancing, (3) Steady-state balancing (dynamic/static), and (4) Fall efficiency and risk.

**Table 1:** Primary results of included studies.

**References**	**Disease**	**Drug and drink information**	**Primary results**	**Quality**
Dai et al. (37)	No diseases affecting balance; No serious illnesses	NR	(1) Significant improvement in the dynamic and static balance of the EG group; (2) Lower and upper limb muscle strength was significantly improved in the EG group;	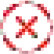
Duan et al. (38)	No diseases affecting balance; Non-acute exacerbations of chronic diseases	NR	(1) Significant increase in EG's Berg balance scale score after 6 months of intervention	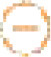
Er (39)	No diseases affecting balance; Non-acute exacerbations of chronic diseases	NR	(1) Berg Balance Scale scores were significantly higher in the EG compared to the control group. (2) Static balance performance of the EG significantly outperformed that of the control group following the intervention.	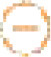
Gao (40)	Older adult in the decline phase	NR	(1) After the 12-week intervention, EG demonstrated a significant improvement in balance ability compared to the CG. (2) EG exhibited a significant reduction in fear of falling compared to CG.	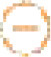
Guan (41)	The post-stroke recovery period	NR	(1) After 3 months, EG demonstrated significant improvements in static balance indices and Berg Balance Scale scores compared to CG.	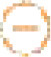
Liu (42)	No diseases affecting balance; Non-acute exacerbations of chronic diseases	NR	(1) In the EG some static balance indices did not show sig nificant improvements compared to the CG. (2) EG's “time up to go text” is a significant improvement over CG.	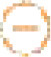
Wu et al. (43)	No diseases affecting balance; Non-acute exacerbations of chronic diseases; Falls have occurred	No drugs affecting mind, vision, gait, balance, etc.	(1) After 30 days of intervention, the fall risk in EG was significantly reduced compared to CG. (2) The balance function of EG is significantly enhanced compared to CG.	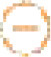
Xie (44)	Disabled	Drinking habits exist in 45.3% of EG and 30.12% of CG	(1) Balance function was significantly improved in EG compared to CG after the intervention	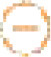
Yao (45)	No diseases affecting balance; Non-acute exacerbations of chronic diseases	NR	(1) EG dynamic and static balance significantly better than CG.	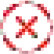
Yu et al. (46)	No diseases affecting balance; Non-acute exacerbations of chronic diseases	NR	(1) Significant improvement in EG fall efficacy compared to CG.	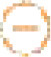
Zhang (47)	No diseases affecting balance; Non-acute exacerbations of chronic diseases	NR	(1) Significant improvement in EG compared to CG static balance. (2) EG's lower body and lumbar strength was significantly improved compared to CG's.	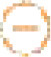
Zhang and Caixia (48)	Stroke-induced hemiplegia;Non-acute exacerbations of chronic diseases	NR	(1) Significant improvement in EG limb function compared to CG after 8 weeks of intervention.	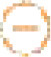
Zhao (49)	No diseases affecting balance; No serious illnesses	NR	(1) EG static balance and lower limb muscle strength significantly improved after 12 weeks of intervention compared to CG. (2) Significant improvement in EG falls efficacy compared to CG after 12 weeks of intervention.	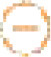
Zheng et al. (50)	Knee Osteoarthritis	Nonsteroidal anti-inflammatory drugs (Loxoprofen Sodium Tablets); Flurbiprofen Cataplasms	(1) BDJ significantly improves dynamic and static balance in KOA patients. (2) Significant improvement in EG falls efficacy compared to CG after 12 weeks of intervention.	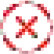
Zhou et al. (51)	The post-stroke recovery period;No serious illnesses	NR	(1) EG demonstrated significant improvements in Berg Balance Scale scores compared to CG.	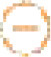
Zhou et al. (52)	No diseases affecting balance; no serious illnesses	NR	(1) EG balance function and lower limb muscle strength improved significantly compared to CG.	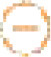
Hua (53)	End-stage renal disease	NR	(1) After 12 weeks, EG demonstrated significant improvements in “Timed Up and Go” and 6 MWT compared to CG.	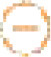
Li et al. (54)	Senile osteoporosis;No serious illnesses	Calcium Carbonate and Vitamin D3 Tablets; Elcatonin Injection	(1) BDJ intervention improved EG balance function compared to controls. (2) BDJ intervention reduces EG fall risk compared to controls.	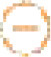
Li (55)	Mild Cognitive Impairment;No diseases affecting balance;No serious illnesses	No drugs affecting brain metabolism and cognitive function.	(1) BDJ significantly enhances the ability to improve static balance in individuals with mild cognitive impairment compared to the CG.	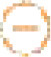
Liao (56)	No diseases affecting balance; Non-acute exacerbations of chronic diseases	NR	(1) Long-term BDJ exercise significantly improves balance function in older women.	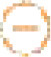
Chen et al. (57)	No diseases affecting balance; Non-acute exacerbations of chronic diseases	NR	(1) Organizing older adult patients to practice group Ba Duan Jin in older adult care homes can effectively reduce the risk of falls	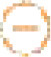
Zhou (58)	Sarcopenia;no serious illnesses	NR	(1) No significant increase in muscle strength in patients with EG's Sarcopenis compared to CG after 12 weeks of intervention	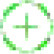
Zhou et al. (59)	Sarcopenia;Non-acute exacerbations of chronic diseases	NR	(1) EG's balance has been significantly improved compared to the CG.	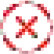
Zhuang et al. (60)	The post-stroke recovery period;No serious illnesses	NR	(1) Adding BDJ to routine rehabilitation enhances balance function in stroke patients.	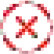
Kuang (61)	Osteoporosis; Non-acute exacerbations of chronic diseases	Calcium Carbonate and Vitamin D3 Tablets	(1) Conventional medication plus BDJ exercise significantly increased Berg balance scale scores	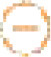
He et al. (62)	No diseases affecting balance; Non-acute exacerbations of chronic diseases	NR	(1) EG demonstrated significant improvements in static balance compared to CG.	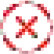
Li (63)	Chronic heart failure;Non-acute exacerbations of chronic diseases	NR	(1) EG demonstrated significant improvements in 6 WMT compared to CG.	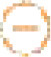
Shi (64)	Falls have occurred; Non-acute exacerbations of chronic diseases	NR	(1) Berg Balance Scale and Modified Falls Efficacy Scale scores of EG patients were significantly better than those of the control group.	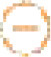
Xu et al. (65)	Coronary heart disease; No diseases affecting balance; Non-acute exacerbations of chronic diseases	Conventional cardiology drug	(1) The test group showed statistically significant improvements in both 6-min walking distance and grip strength after the intervention compared to the control group.	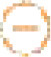
Wang et al. (66)	Psychiatric disorder; Non-acute exacerbations of chronic diseases	NR	(1) Significantly improved balance function and fall prevention confidence in EG compared to CG.	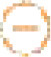
Carcelén-Fraile et al. (67)	No diseases affecting balance; Non-acute exacerbations of chronic diseases	No drugs that impaired the central nervous system, co-ordination, or balance (e.g., anxiolytics, antidepressants, or vestibular sedatives).	(1) 12 weeks of BDJ training benefits muscle strength and postural control.	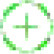
Liu et al. (68)	No diseases affecting balance; Non-acute exacerbations of chronic diseases	NR	(1) Balance function was significantly improved post-intervention compared to pre-intervention. (2) Confidence in coping with falls was significantly improved post-intervention compared to pre-intervention.	NA
Xiao et al. (69)	Type Diabetes Mellitus;	NR	(1) A 6-month Baduanjin intervention significantly improved EG balance compared to CG.	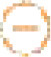
Xiao and Zhuang (70)	Mild to moderate Parkinson; Non-acute exacerbations of chronic diseases	NR	(1) The Baduanjin intervention significantly improved Berg balance scale score and 6 WMT compared to the CG.	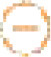
Ye et al. (71)	Knee Osteoarthritis; No serious illnesses	No drugs that could affect the musculoskeletal system or postural stability (e.g., antidepressants, dopaminergic agents, and hypnotics).	(1) The perimeter and ellipse area with both open- and closed-eyes conditions were significantly improved at week eight in the experimental group. (2) The ellipse area with open-eyes condition, WOMAC index, and stiffness and physical function domains were significantly decreased after the 12 weeks of Baduanjin training compared to the CG, and only the perimeter area with both open- and closed-eyes conditions was not statistically significant at week 12 in the EG.	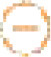
Yuen et al. (72)	Chronic Stroke; No serious illnesses	NR	(1) After 16 weeks of intervention, overall balance was significantly improved compared to CG. (2) Stability limits and fall efficacy showed no significant improvement after the 16-week intervention.	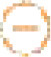
Ye et al. (73)	Post-Stroke cognitive impairment; No serious illnesses	No alcohol or drug abuse	(1) After the 24-week intervention, Baduanjin group exhibited significantly better FMA, BBS results than the control group. (2) Baduanjin exercise group showed significant improvements in spatial gait.	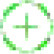
Ye et al. (74)	Knee Osteoarthritis; No serious illnesses	No drugs that could affect the musculoskeletal system or proprioception and postural stability (e.g., antidepressants, dopaminergic agents, and hypnotics).	(1) For postural stability at the anterior-posterior direction with eyes closed, Baduanjin Qigong group showed significant improvement compared to controls after the 12 weeks of intervention.	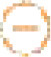
Tou et al. (75)	Frailty status; No diseases affecting balance	NR	(1) After the 4-month intervention, there were no significant differences in fall efficacy, TUG scores, and 30s sit-to-stand in the intervention group compared to the CG.	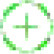
Jiao (76)	No diseases affecting balance; No serious illnesses	NR	(1) The Baduanjin intervention had a significant effect on the dynamic balance of retired teachers.	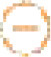

Fair, red goal; Good, yellow goal; Excellent, green goal; NR, not report; NA, not applicable.

### 3.3 Methodological quality of included studies

A quasi-experimental study was excluded from the quality assessment (68). The remaining 39 studies underwent quality evaluation, with 6 rated as fair (37, 45, 50, 59, 60, 62), 29 as good (38–44, 46–49, 51–57, 61, 63–66, 69–72, 74, 76), and 4 classified as excellent (58, 67, 73, 75). Overall, the majority of the included literature was of good quality. The detailed results of the quality assessment are provided in Appendix C.

### 3.4 The effect of Baduanjin on balanced performance

**Figure 2:**
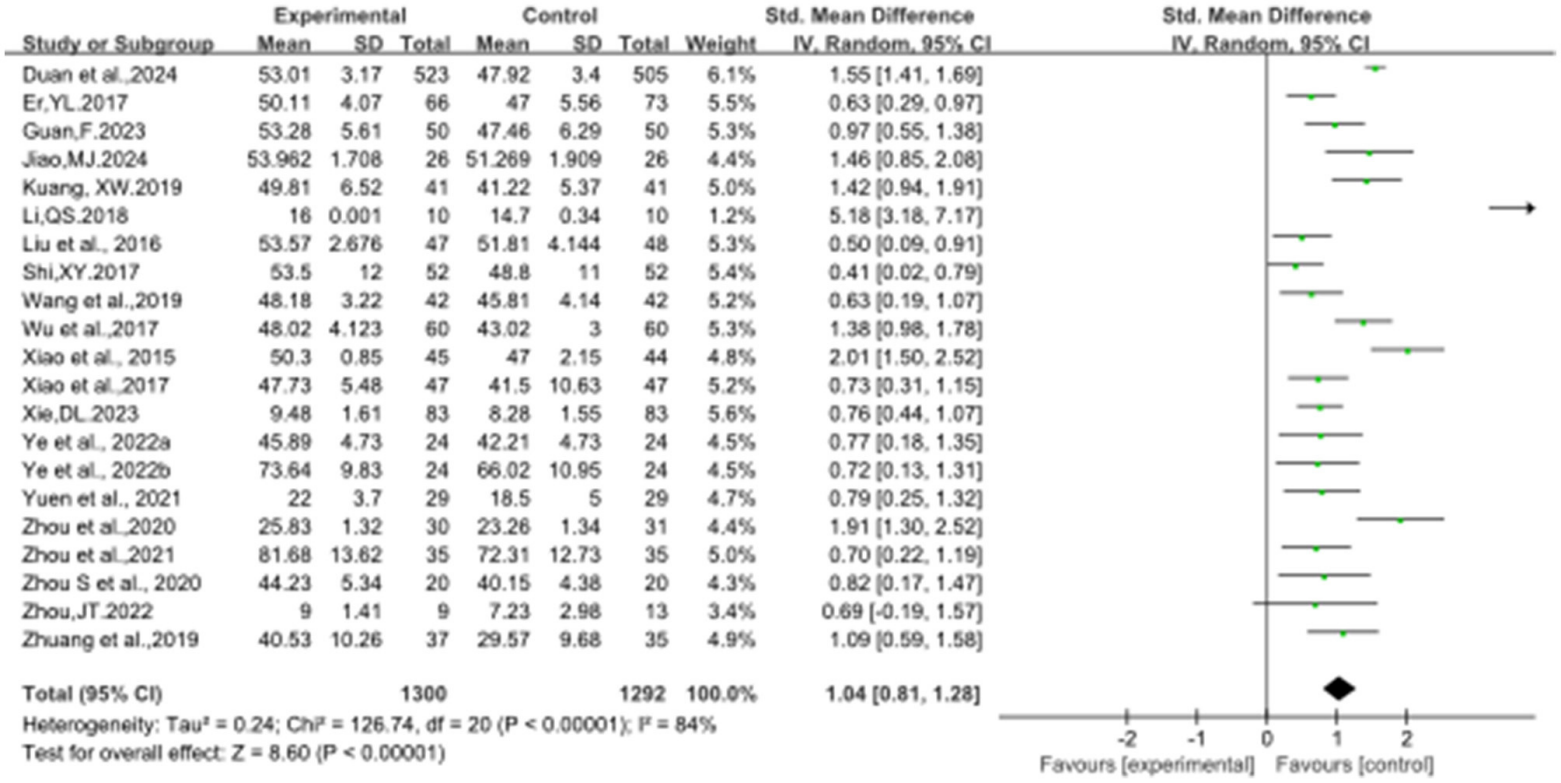
BTB forest plot.

**Figure 3:**
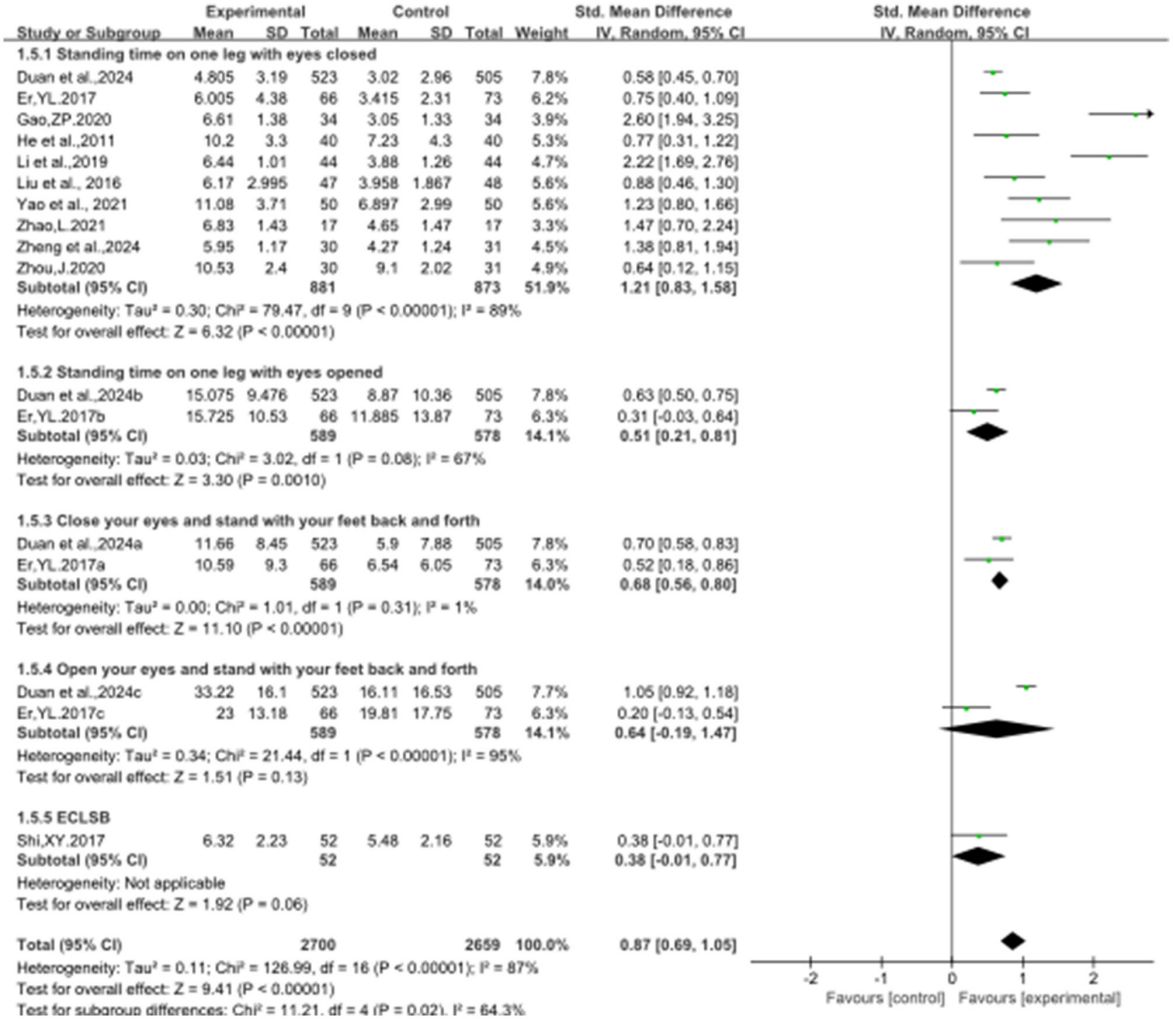
SBT forest plot.

**Figure 4:**
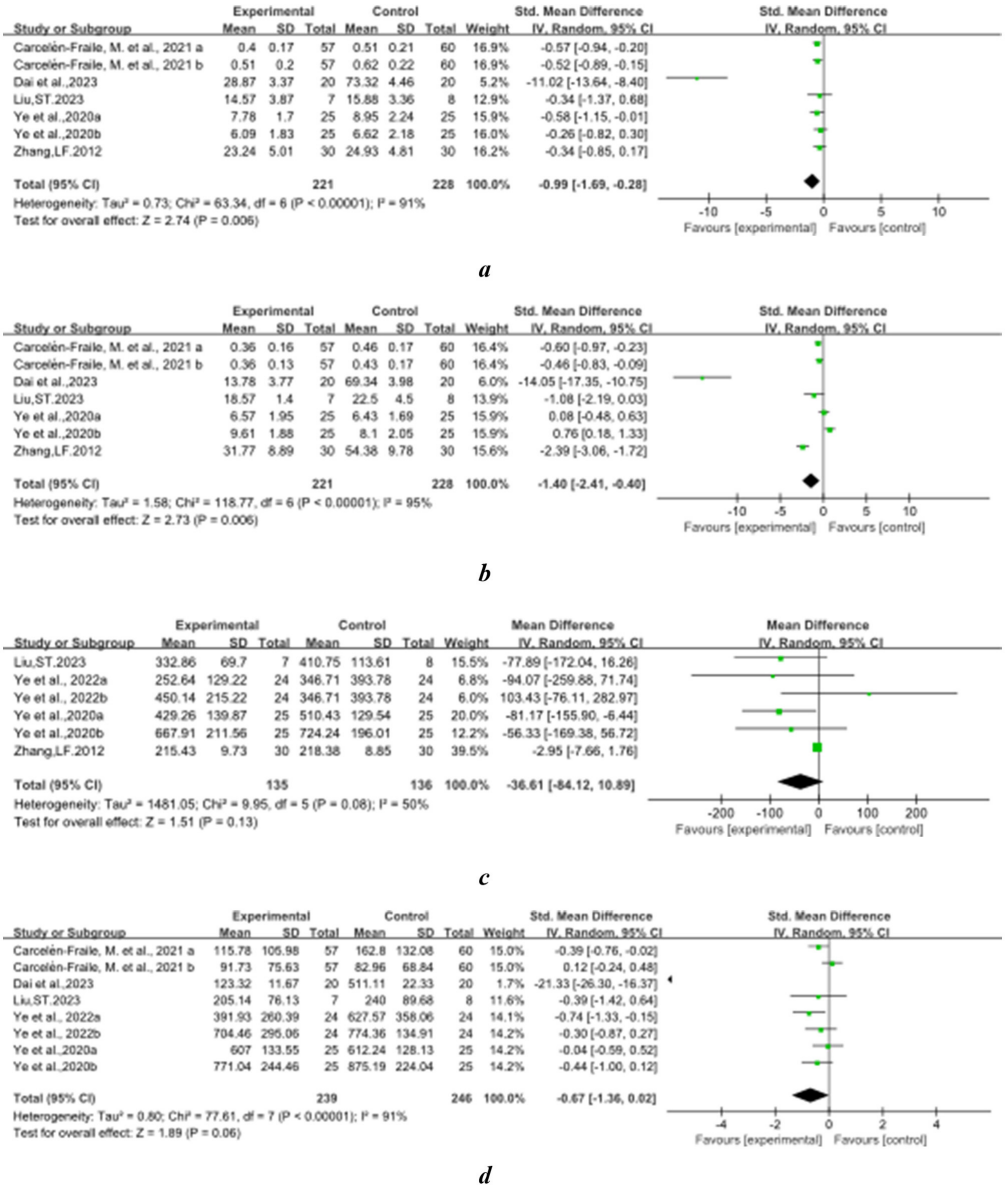
SBT related parameters. **(A)** Displacement distance in the left and right directions of the cop; **(B)** displacement distance in front and back direction of cop; **(C)** cop track length; **(D)** area of the cop encircling ellipse.

**Figure 5:**
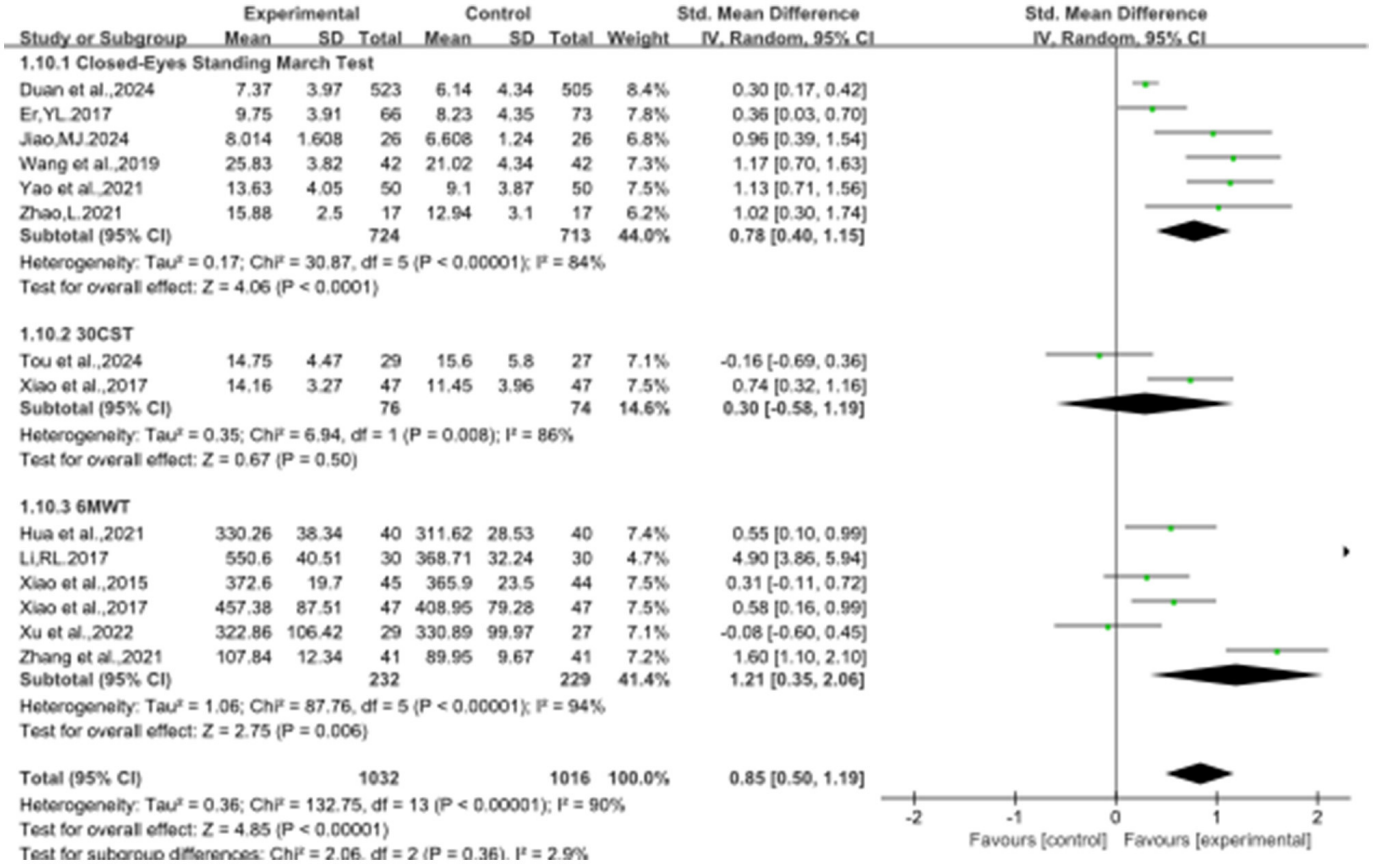
DBT forest plot.

**Figure 6:**
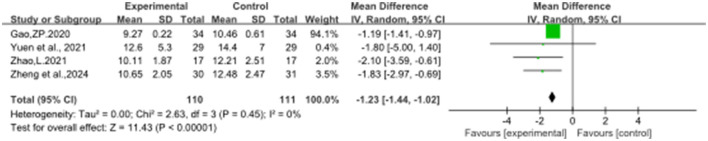
Five times sit to stand test forest plot.

**Figure 7:**
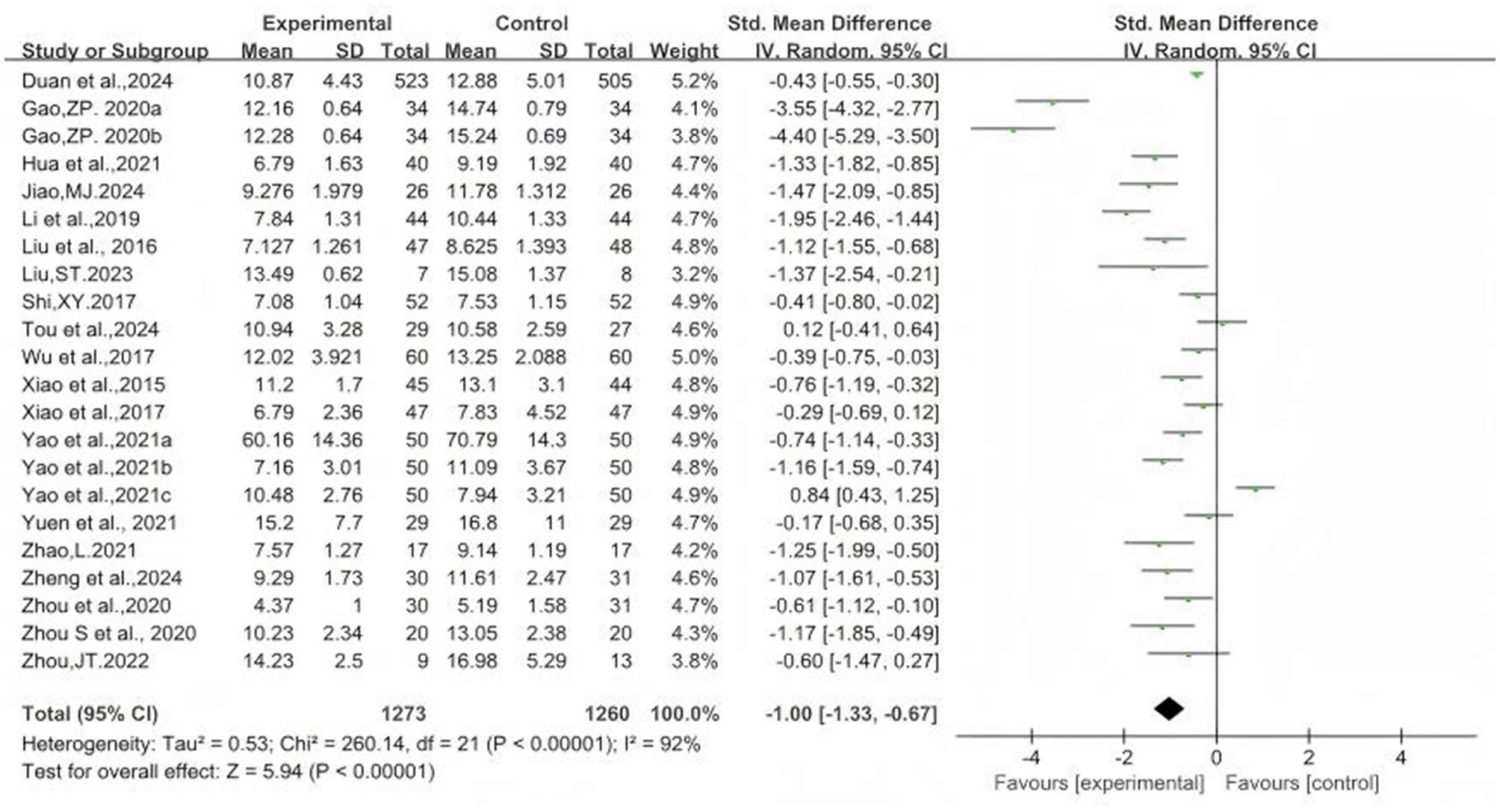
PBT forest plot.

**Figure 8:**
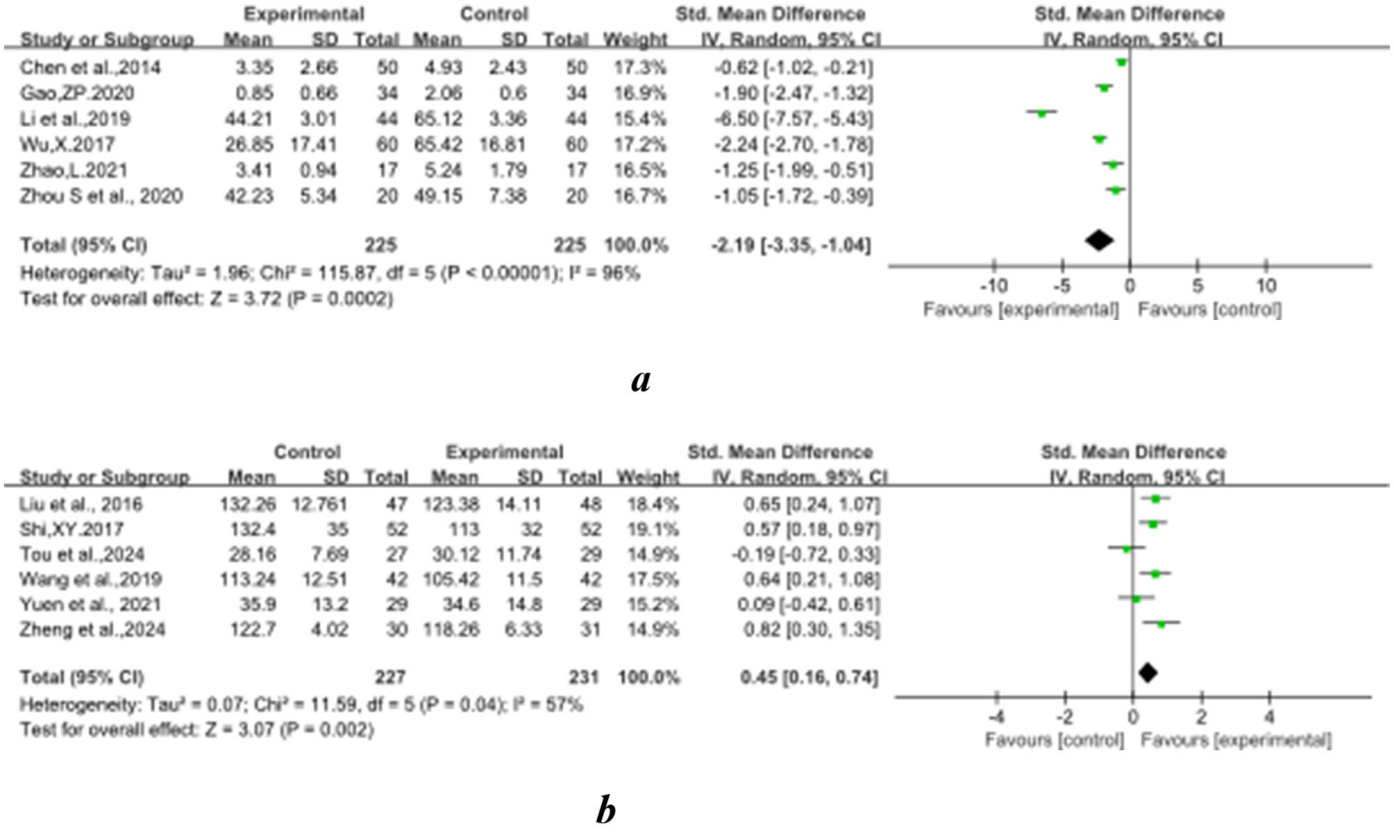
Falls-related indicators forest plot. **(A)** Fall risk. **(B)** Fall efficacy.

Figures 2–8 illustrate the effects of BDJ on balance performance and fall risk among older adults. The results indicated that BDJ had a significant positive effect on static balance compared to the control group (SMD = 0.87, 95% CI: 0.69–1.05, *P* < 0.01, df = 16), with substantial improvements in one-legged standing [eyes open (OE): SMD = 1.21, 95% CI: 0.83–1.58, *P* < 0.01, df = 9; eyes closed (CE): SMD = 0.51, 95% CI: 0.21–0.81, *P* = 0.001, df = 1] and anterior-posterior standing with CE (SMD = 0.68, 95% CI: 0.56–0.80, *P* < 0.01, df = 1). However, no significant improvement was observed in anterior-posterior standing with OE. The analysis of static balance parameters revealed a significant reduction in COP displacement distance (left-right: SMD = −0.99, 95% CI: −1.69–−0.28, *P* < 0.01, df = 7; anterior-posterior: SMD = −1.40, 95% CI:−2.41–−0.40, *P* < 0.01, df = 6), although COP trajectory length and COP ellipse area did not show significant changes. BDJ also significantly improved dynamic balance (SMD = 0.85, 95% CI: 0.50−1.19, *P* < 0.01, df = 13). Specifically, a fixed-effects model analysis of five sit-to-stand tests demonstrated a notable reduction in time (MD = −1.23, 95% CI: −1.44–−1.02, *P* < 0.001, df = 3). Proactive balance also showed significant improvement compared to the control group (SMD = −1.00, 95% CI: −1.33–−0.67, *P* < 0.001, df = 21). In addition, the study analyzed fall-related outcomes. Following the BDJ intervention, participants exhibited significant reductions in fall risk (SMD = −2.19, 95% CI: −3.35 to −1.04, *P* < 0.001, df = 5) and improvements in fall efficacy (SMD = 0.45, 95% CI: 0.16–0.74, *P* < 0.001, df = 5).

Furthermore, the impact of BDJ in comparison to various control groups was evaluated (Appendix D), demonstrating that BDJ exhibited a significant superiority over health education, routine care or rehabilitation, regular activities, and walking for diverse balance abilities in older. Despite the observation of no significant advantage of BDJ over routine care or rehabilitation in static balance (SMD = 0.69, 95%CI: −0.23–1.61, *P* = 0.14, df = 2), the overall evidence suggests that BDJ practice outside of essential activities significantly enhances balance in older adults and is more efficacious than walking.

### 3.5 Optimal parameters for balanced performance in Baduanjin

**Figure 9:**
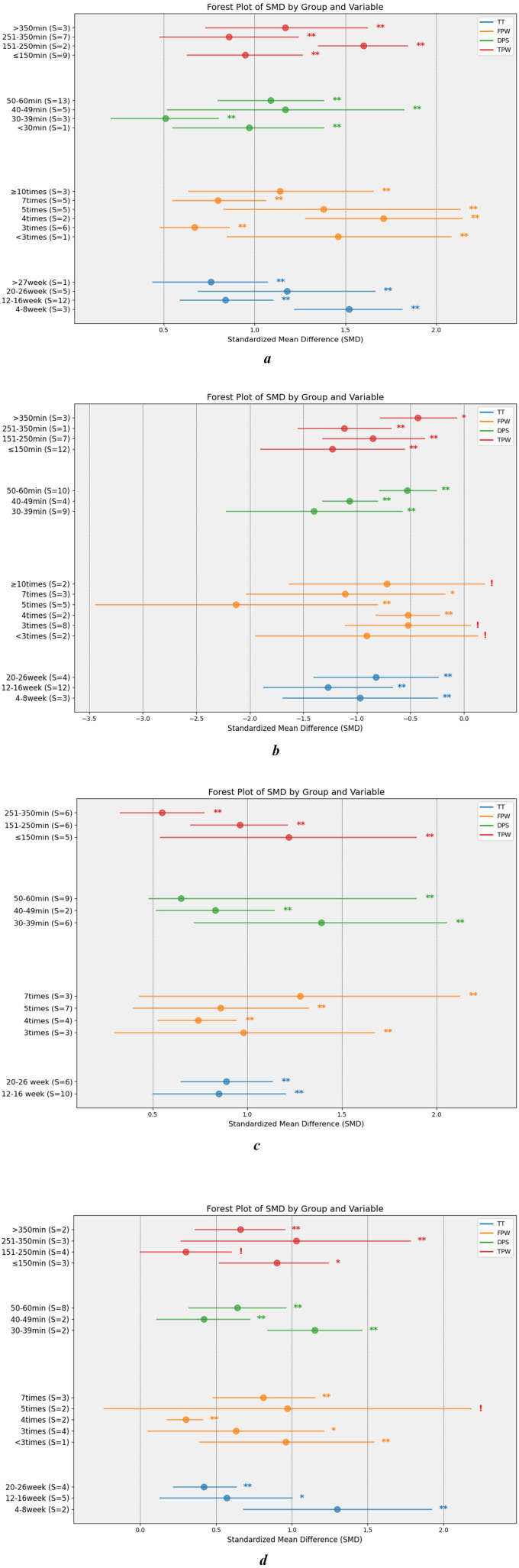
Dose subgroup results. BTB, static balance indicators; PBT, Proactive balancing indicators; SBT, static balance indicators; DBT, dynamic balance indicators; TT, The total number of weeks during which the subject engaged in physical exercise; FPW, The number of times per week that subjects participated in physical activity; DPS, Time duration during which subjects were engaged in physical activity; TPW, Total time spent by subjects in physical activity per week; S, sample size. **P < 0.01; *P < 0.05; ^!^*P* ≥ 0.05. **(A)** BTB. **(B)** PBT. **(C)** SBT. **(D)** DBT.

Subgroup analyses were conducted to examine dose-related parameters, revealing a reduction in heterogeneity to varying degrees. The relevant data metrics are provided in Appendix E. Figure 9 displays the effect sizes and 95% confidence intervals for data points demonstrating significant effects.

#### 3.5.1 Total training time

Subgroup analyses revealed that the optimal timeframe for DBT was 4–8 weeks (SMD = 1.30, 95%CI: 0.68–1.92, *P* < 0.01, df = 1), during which its effects were most pronounced. Notably, the efficacy observed at 12–16 weeks was comparable to that seen at 20–26 weeks. For SBT, training during weeks 20–26 (SMD = 0.89, 95% CI: 0.65–1.13, *P* < 0.001, df = 5) yielded only marginally better results than weeks 12–16 (SMD = 0.85, 95% CI: 0.50–1.20, *P* < 0.001, df = 9). PBT showed its highest improvement during weeks 12–16 (SMD = −1.27, 95% CI: −1.87–−0.67, *P* < 0.001, df = 11), with a decline in effectiveness observed by weeks 20–26. The BTB showed the greatest gains during weeks 4–8 (SMD = 1.52, 95% CI: 1.22–1.81, *P* < 0.001, df = 2). An upward trend in BTB effectiveness was noted from weeks 12–16 (SMD = 0.84, 95% CI: 0.59–1.10, *P* < 0.001, df = 12) to weeks 20–26 (SMD = 1.18, 95% CI: 0.69–1.66, *P* < 0.001, df = 4).

#### 3.5.2 Training frequency

Interventions administered seven times per week (SMD = 1.28, 95% CI: 0.43–2.12, *P* < 0.001, df = 2) yielded the most significant improvements in SBT, while no substantial differences were observed between 3, 4, and 5 weekly sessions. For DBT, seven training sessions per week (SMD = 0.81, 95% CI: 0.48–1.15, *P* < 0.001, df = 2) produced the best results, with three sessions being slightly less effective. Optimal performance in PBT was achieved with 5 weekly sessions (SMD = −2.13, 95% CI: −3.44–−0.81, *P* < 0.01, df = 4), with effectiveness declining at seven sessions per week. BTB results were most favorable with 4 weekly sessions (SMD = 1.71, 95% CI: 1.28–2.14, *P* < 0.01, df = 1), although this finding was based on only two studies. The second-highest gains were observed with 5 weekly sessions (SMD = 1.38, 95% CI: 0.63–2.13, *P* < 0.01, df = 4).

#### 3.5.3 Duration of training per time

The greatest improvements in SBT (SMD = 1.39, 95% CI: 0.72–2.05, *P* < 0.001, df = 5) and PBT (SMD = −1.40, 95% CI: −2.22–−0.58, *P* < 0.001, df = 8) were observed with training sessions lasting 30–39 min, with a gradual decline in effectiveness as session duration increased. For DBT, the optimal results were achieved with 50–60 min sessions (SMD = 0.64, 95% CI: 0.32−0.96, *P* < 0.001, df = 7), while BTB saw the highest benefits with 40–49 min sessions (SMD = 1.17, 95% CI: 0.52−1.82, *P* < 0.001, df = 4). Training sessions lasting 50–60 min (SMD = 1.09, 95% CI: 0.80–1.38, *P* < 0.001, df = 12) provided slightly lower gains in BTB compared to the 40–49 min range.

#### 3.5.4 Training duration per week

The maximum improvements in SBT (SMD = 1.22, 95% CI: 0.54–1.89, *P* < 0.001, df = 4) and proactive balance (PB) performance (SMD = −1.23, 95% CI: −1.90–−0.56, *P* < 0.001, df = 10) were achieved with a relatively low total training time (≤150 min). In contrast, optimal performance in dynamic balance training (DBT) was observed with weekly training totals in the range of 251–350 min (SMD = 1.03, 95% CI: 0.27–1.78, *P* < 0.001, df = 2). For the balance test battery (BTB), the most effective training time was between 151 and 250 min (SMD = 1.60, 95% CI: 1.35–1.84, *P* < 0.001, df = 2), reflecting significant gains across both time periods.

### 3.6 Moderator analysis

The study underwent moderated factor analysis (Table 2), incorporating covariates such as the control group, health status, age, and dose-related parameters. Two studies were excluded from the regression analysis due to the lack of dose parameters (45, 63). The results of the multivariate modeling indicated that the frequency of exercise sessions per week significantly moderated the positive effects on SBT (β = 2.538, *P* = 0.046). Additionally, the type of control group demonstrated a significant overall moderating effect (β = 0.363, *P* < 0.01). Univariate modeling revealed potential moderating effects of the control group on PBT, SBT, DBT, and overall balance outcomes. Health status was found to significantly moderate SBT (β = 0.664, *P* = 0.005). Furthermore, the duration of each training session significantly influenced PBT (β = −0.539, *P* = 0.002), SBT (β = −0.571, *P* = 0.01), and overall balance (β = −0.249, *P* = 0.042).

**Table 2:** Moderator analysis.

	**BTB**		**PBT**		**SBT**		**DBT**		**All**	
	β	***P***	β	***P***	β	***P***	β	***P***	β	***P***
**Multivariate model**										
Control group	0.306	0.797	0.770	0.058	0.335	0.748	1.147	0.141	0.363	<0.01^**^
Health status	0.264	0.555	0.054	0.890	0.697	0.264	0.178	0.717	0.111	0.405
Age	0.123	0.855	0.412	0.387	0.21	0.754	−0.277	0.665	0.330	0.109
TT	−0.122	0.872	0.138	0.675	−0.006	0.987	−0.212	0.752	−0.029	0.821
FPW	−0.063	0.969	0.856	0.402	2.538	0.046^*^	2.154	0.132	0.176	0.709
DPS	−0.196	0.868	−0.811	0.206	2.126	0.079	2.100	0.115	−0.401	0.225
TPW	0.050	0.978	−1.077	0.355	−2.728	0.063	−1.762	0.178	−0.187	0.710
**Univariate model**										
Control group	0.266	0.057	0.307	0.04^*^	0.621	0.004^**^	0.525	0.009^**^	0.202	<0.01^**^
Health status	0.278	0.144	0.126	0.348	0.664	0.005^**^	0.239	0.30	0.150	0.121
Age	0.055	0.852	0.393	0.117	0.144	0.683	0.128	0.680	0.304	0.055
TT	−0.114	0.536	−0.144	0.429	0.228	0.457	−0.393	0.274	−0.139	0.190
FPW	0.079	0.586	0.089	0.627	0.302	0.188	0.289	0.341	0.145	0.117
DPS	0.084	0.671	−0.539	0.002^**^	−0.571	0.01^*^	0.072	0.838	−0.249	0.042^*^
TPW	0.109	0.425	−0.136	0.370	−0.326	0.181	0.235	0.287	0.007	0.951

BTB, static balance indicators; PBT, Proactive balancing indicators, SBT, static balance indicators; DBT, dynamic balance indicators; TT, The total number of weeks during which the subject engaged in physical exercise; FPW, The number of times per week that subjects participated in physical activity; DPS, Time duration during which subjects were engaged in physical activity; TPW, Total time spent by subjects in physical activity per week.

* *P* < 0.05;

** *P* < 0.01.

### 3.7 Sensitivity analysis

The study conducted a sensitivity analysis on the combined data, and the results remained robust after sequentially presenting each study, confirming the robustness of the findings. The details of the sensitivity analysis are provided in Appendix F.

### 3.8 Publication bias and correction

The funnel plot indicated the presence of publication bias in groups with combined data of seven or more studies (Appendix G). The funnel plots for DBT and BTB appeared less symmetrical; however, only Egger's test indicated significant asymmetry for DBT (*P* = 0.027), and Begg's test (*P* = 0.090 and *P* = 0.053) indicated no significant asymmetry. In contrast, the funnel plots for SBT showed significant asymmetry, as suggested by Egger's test (*P* = 0.215), while Begg's test (*P* = 0.003) indicated potential publication bias. To address this, we corrected the effect size for static balance using the Trim and Fill method proposed by Duval and Tweedie (79), resulting in a Hedges' g of 0.731 (95% CI: 0.526–0.936). The funnel plot for PBT exhibited slight asymmetry, as confirmed by Egger's test (*P* = 0.022) and Begg's test (*P* = 0.022), suggesting possible publication bias. This adjustment added seven data points, resulting in a corrected effect size of Hedges' g = −0.495 (95% CI: −0.850–−0.139).

## 4 Discussion

The present study reviewed the effects of BDJ on balance and fall-related indices in older adults, highlighting the impact of training parameters across different balance abilities. The findings indicate that: (1) BDJ significantly enhances overall balance in older adults, with proactive balance training yielding greater improvements compared to homeostatic balance (both dynamic and static); (2) Notable benefits begin to emerge after a minimum of 12 weeks of training, with 5–7 practice sessions per week lasting 30–49 min identified as the optimal training duration; (3) BDJ training significantly reduces fall risk and improves fall efficacy, thereby increasing older adults' confidence in managing potential falls.

### 4.1 Effect of Baduanjin on balanced performance

BDJ was associated with significant improvements in homeostatic balance, proactive balance, and balance test battery performance among older adults. However, no passive balance-related metrics were available for analysis. Previous research has established that balance is an idiosyncratic task (80), with one study noting a lack of strong correlation between different balance abilities in middle-aged and older adults (81). This indicates that comprehensively improving balance ability is crucial for non-specific exercise tasks. BDJ exercise is a mind-body practice akin to Tai Chi, incorporating stretching and frequent shifts of the center of gravity in various directions. Unlike idiosyncratic balance training, the enhancements achieved through BDJ are rooted in its kinematic characteristics. Both approaches to improving balance are based on the interplay between training adaptations and neural plasticity, with exercises promoting task-specific neural adaptations (82). Balance is primarily maintained through the synergy of the proprioceptive, visual, and vestibular systems (80). However, age-related degenerative changes in these systems lead to declines in sensory, central nervous, and skeletal musculature functions, resulting in reduced balance ability, irrespective of pathological dysfunction (83).

Prior research has shown that Baduanjin enhances proprioception in muscles and joints, particularly in the lower extremities, contributing to improved postural control and balance stability (21, 74). Proprioception plays a crucial role in postural control, especially during postural orientation, by providing the central nervous system with feedback about the position and movement of the body in space (84). BDJ strengthens proprioception by shifting the center of gravity across the horizontal, coronal, and sagittal planes at high frequencies, with a particular emphasis on the alignment of body and mind, which is employed to perceive the movement of forces in diverse directions, as well as the folding and rotation of bodily components. This aligns with the objectives of proprioceptive training, which aims to enhance sensory perceptions such as positional awareness and movement sensation (85). This type of slow-moving physical activity, which integrates both physical and mental exercise, has significantly improved balance in older adults (86). Additionally, certain movements challenge the visual and vestibular systems, enhancing their functions through habituation exercises. For instance, in the fifth form, “Shaking the Head and Swinging the Tail to Eliminate the Heart Fire,” the head is lowered while the center of gravity shifts, requiring rotation around the central axis via the neck muscles. This movement increases the difficulty for the vestibular system in spatial perception and balance maintenance. Approximating perturbation training has improved control of disturbed balance and reorganize feedback responses (87). It is possible that such training leads to increased adaptations in the spinal cord and cerebral cortex, potentially enhancing the automation of postural control (82). Furthermore, performing BDJ involves repetitive movements guided by musical instructions, along with synchronized breathing to enhance mental focus. The multiple perturbation strategy, which has optimized sensory integration, has significantly facilitated the development of balance, especially in the realm of proactive balance, among older adult (88). This likely accounts for the superior effects of BDJ on active balance compared to homeostatic balance, as greater gains are observed in dynamically complex tasks. However, it is important to note that publication bias exists in the PBT, with an adjusted effect size indicating a suboptimal result. Caution is warranted when interpreting these findings.

### 4.2 Optimal training parameters for Baduanjin

A study by Wang et al. demonstrated that the optimal dose of 24-Style Simplified Tai Chi Chuan (24-STC) for improving balance in older adults was 45–60 min, four times per week, over a minimum of 8 weeks (78). This aligns with the findings of our study, as BDJ and Tai Chi share similar kinesiological principles and cultural roots. However, in terms of session duration and training weeks, our study found that BDJ requires less time per session but longer overall training duration, suggesting that high-frequency and extended BDJ practice may offer greater benefits for balance in older adults. While both BDJ and 24-STC are mind-body exercises, BDJ engages more sensory systems. For instance, certain movements where the head is positioned below waist level (e.g., in the 5th and 6th forms) are more challenging than comparable Tai Chi movements. This is consistent with our findings, where improvements in static balance with eyes closed were superior to those with eyes open, likely due to the enhanced activation of motor-sensory systems.

The intensity of BDJ exercise for healthy adults aged 18–64 has been estimated at 3.2 MET, classifying it as moderate-intensity activity according to the Chinese Society of Sports Science (89). The U.S. Centers for Disease Control and Prevention recommends that older adults engage in at least 150 min of moderate-intensity activity per week, which 30 min per day, 5 days per week. Our study builds on this by finding that BDJ may require an even higher frequency of exercise to optimize balance improvements. Specifically, we observed balanced improvements in different balance abilities after 12 weeks of training. However, our data is limited by the small number of short-term (*n* ≤ 2) and long-term studies. In terms of weekly training frequency, 5–7 sessions per week yielded the most balanced development across different balance skills, though our study lacked data on higher frequencies (*n* ≥ 10), so we cannot exclude the possibility of additional benefits at higher frequencies. Interestingly, no significant moderating effect of training frequency on balance improvements was identified. Regarding the number of weeks, both 12–16 weeks and 20–26 weeks of training showed improvements across various balance metrics, with only slight differences between the two periods. However, improvements in proactive balance (PB) and dynamic balance (DB) were not significant in the 4–8 week range, although only two trials were included in this analysis.

This study examined the effect of exercise duration on outcomes, finding that optimal results were achieved with session lengths of 30–49 min, while a decline in proactive balance (PB) performance was observed at 50–60 min. Given the greater impact of BDJ on PB, it may not be advisable to extend practice sessions to 50–60 min. According to video tutorials published by the General Administration of Sport of China (GASPC), a standard BDJ session lasts approximately 12 min. Intervention protocols that incorporate 2–3 BDJ cycles after a preparatory activity appear to yield higher balance gains. Unfortunately, only one study included an intervention session shorter than 30 min (a single 15-min practice session), yet even this shorter session showed significant post-intervention improvements in the Berg Balance Scale (BBS) compared to both pre-intervention levels and the control group (41).

### 4.3 Effect of moderator factors on balance performance

The findings of this systematic review and meta-analysis indicate that neither the number of weeks of exercise nor the weekly duration significantly moderated the effect on balance outcomes. However, as previously mentioned, high-frequency exercise was recommended, and regression analysis results confirmed that the frequency of weekly training sessions and the type of control group had a significant impact on effect size. The control group's role may stem from incorporating participants' daily activities (e.g., walking) or essential tasks (e.g., routine care of sick individuals), which could influence balance performance. However, subgroup analyses revealed that BDJ outperformed the majority of control interventions across various balance abilities. After adjusting for variables, factors such as age, health status, and session duration significantly moderated balance outcomes. These findings align with prior research, suggesting that high-frequency training combined with appropriate session durations enhances the benefits, especially when considering total intervention time (22). Nevertheless, our study underscores the importance of carefully regulating the duration of each session. Prolonged sensory integration may lead to partial sensory deprivation, potentially causing sensory overload (90), as the continuous processing of sensory information can induce physical and mental fatigue earlier (91). Adverse effects such as dizziness, fatigue, palpitations, and chest tightness have been documented in safety checks related to BDJ practice (24). A key study that examined this phenomenon involved 90-min sessions in patients with chronic fatigue syndrome, where the extended duration may have contributed to adverse effects (92). This suggests that excessively long BDJ sessions could act as a trigger for such negative outcomes.

### 4.4 Research gap

While the outcomes of the majority of the included studies and the findings of this review are indeed encouraging, the overall quality of the research leaves something to be desired. The current body of work on Baduanjin (BDJ) and its impact on balance function has primarily concentrated on interventions spanning from 12 to 26 weeks. However, there is a notable absence of studies focusing on acute interventions and those with a long-term perspective. Additionally, the sustainability of the benefits post-intervention is an area that requires further investigation, yet only a few studies have conducted follow-up assessments. Regarding the outcomes, although most functional tests have yielded favorable results, there is an urgent need to enhance the precision of balance measurements. For instance, incorporating the use of pressure transducers to capture parameters of postural control is crucial, as the current data in this regard are less than optimal. In summary, underscore the imperative for future investigations to concentrate on several key areas: (1) enhancing the methodological rigor of research, (2) extending follow-up periods to discern the lasting impacts of BDJ on balance, (3) refining the accuracy of balance metrics, and (4) undertaking both long-term observational studies and acute intervention trials among specific populations.

## 5 Limitations

It is important to acknowledge several limitations of this study: (1) During the subgroup analyses, the absence of short-term and long-term studies limited the ability to assess short-term benefits and reduced the reliability of long-term outcomes. (2) Heterogeneity was observed among some subgroups. While potential sources of heterogeneity were explored, focusing solely on dose likely diminished the precision of the analysis. (3) Some balance metrics exhibited potential publication bias, and although adjustments were made, this may have affected the interpretation of the findings. Additionally, the study lacked a sufficient number of high-quality studies to establish a more solid basis for its conclusions. (4) The moderated effects analysis faced challenges, including data loss and difficulties in accurately categorizing health status due to incomplete information in the primary studies. Moreover, most studies did not provide the mean age for the entire sample, requiring the use of weighted conversions, which introduced some level of imprecision. (5) The lack of proactive balance data further limited the ability to draw comprehensive conclusions regarding overall balance outcomes.

## 6 Conclusions

In conclusion, this systematic review and meta-analysis suggest that BDJ is an effective intervention for enhancing balance and preventing falls in older adults that could be more beneficial than routine care, walking or regular activities for improving balance and reducing the risk of falls in older adults. For optimal balance outcomes, it is recommended to engage in a minimum of 12 weeks of training, consisting of five to seven sessions of 30 to 39 min each week. This dosage has been shown to yield significant improvements across various balance metrics, a finding that is consistently supported by the majority of studies included in this review.

However, exceeding this duration in a single session may diminish the observed benefits. Caution is advised in interpreting these results due to limitations related to heterogeneity, study quality, and the overall number of studies included.

## Data Availability

The original contributions presented in the study are included in the article/Supplementary material, further inquiries can be directed to the corresponding author.
