# Supplementary material for: Optimal exercise parameters of Baduanjin for balance in older adults: a systematic review and meta-analysis
Source: Front Public Health. 2025 Mar 19;13:1541170. doi: 10.3389/fpubh.2025.1541170 (PMC11961421; doi:10.3389/fpubh.2025.1541170)
Supplement: Supplementary file 1 [file Data_Sheet_1.pdf]

Appendix

A: Search strategies for Chinese and English databases

English database

Pubmed

#5 (((#1) AND (#2)) AND (#3)) AND (#4)

#4 ((((("randomized controlled trial"[Title/Abstract]) OR ("controlled trial"[Title/Abstract]))OR ("randomised controlled study"[Title/Abstract])) OR (controlled[Title/Abstract])) OR (randomized[Title/Abstract])) OR ("control group"[Title/Abstract])) OR (placebo[Title/Abstract])

#3 ((((((((((balance) OR ("body equilibrium")) OR (equilibrium)) OR ("postural balance")) OR ("postural control")) OR ("postural equilibrium")) OR (falling)) OR (accidental falls)) OR ("slip and fall")) OR (musculoskeletal equilibrium)) OR (fall)

#2 (((Baduanjin) OR ("eight section brocades")) OR (qigong)) OR ("Traditional Chinese exercise")

#1 (((((Old) OR (senior)) OR (aged)) OR (elderly)) OR (middle aged)) OR (elder)

Embase

(old OR senior OR 'aged'/exp OR 'aged' OR 'aged patient' OR 'aged people' OR 'aged person' OR 'aged subject' OR 'elderly' OR 'elderly patient' OR 'elderly people' OR 'elderly person' OR 'elderly subject' OR 'senior citizen' OR 'senium' OR 'middle aged'/exp OR elder OR aged OR 'middle aged') AND ('baduanjin'/exp OR 'eight section brocades' OR 'qigong exercise'/exp OR 'traditional chinese exercise'/exp OR baduanjin OR qigong OR 'traditional chinese exercise') AND ('balance'/exp OR 'body equilibrium'/exp OR 'body sway' OR 'equilibrium, body' OR 'musculoskeletal equilibrium' OR 'postural balance' OR 'postural equilibrium' OR 'postural control'/exp OR 'falling'/exp OR 'fall' OR 'falling' OR 'slip and fall' OR balance OR fall OR 'body equilibrium' OR falling OR 'postural control' OR 'accidental falls') AND ('randomized controlled trial'/exp OR 'controlled trial, randomized' OR 'randomised controlled study' OR 'randomised controlled trial' OR 'randomized controlled study' OR 'randomized controlled trial' OR 'trial, randomized controlled' OR 'control group'/exp OR controlled:ti OR randomized:ti OR controlled:ab OR 'randomized controlled trial':ti)

Web of Science

#1: (((((ALL=(old)) OR ALL=(senior)) OR ALL=(aged)) OR ALL=(elderly)) OR ALL=(middle aged)) OR ALL=(elder)

#2: (((ALL=(baduanjin)) OR ALL=(eight section brocades)) OR ALL=(qigong)) OR ALL=(Traditional Chinese exercise)

#3: ((((((((((ALL=(balance)) OR ALL=(body equilibrium)) OR ALL=(equilibrium)) OR ALL=(postural balance)) OR ALL=(postural control)) OR ALL=(postural equilibrium)) OR

ALL=(falling)) OR ALL=(accidental falls)) OR ALL=(slip and fall)) OR ALL=(musculoskeletal equilibrium)) OR ALL=(fall)

#4: ((((((TS=(randomized controlled trial)) OR TS=(controlled trial)) OR TS=(randomized controlled study)) OR TS=(controlled)) OR TS=(randomized)) OR TS=(control group)) OR TS=(placebo)

#5: #1AND #2 AND #3AND #4

Google Scholar (top 200 items)

(“old” OR “senior” OR “aged” OR “elderly” OR “middle aged” OR “elder”) AND (“baduanjin” OR “eight section brocades” OR “qigong” OR “Traditional Chinese exercise”) AND (“balance” OR “body equilibrium” OR “equilibrium” OR “postural balance” OR “postural control” OR “postural equilibrium” OR “falling” OR “accidental falls” OR “slip and fall” OR “musculoskeletal equilibrium” OR “fall”) AND (“randomized controlled trial” OR “controlled trial” OR “randomised controlled study” OR “controlled” OR “randomized” OR “control group” OR “placebo”)

Cochrane Library

(old OR senior OR aged OR elderly OR middle aged OR elder) AND (baduanjin OR eight section brocades OR qigong OR Traditional Chinese exercise) AND (balance OR body equilibrium OR equilibrium OR postural balance OR postural control OR postural equilibrium OR falling OR accidental falls OR slip and fall OR musculoskeletal equilibrium OR fall) AND (randomized controlled trial OR controlled trial OR randomized controlled study OR controlled OR randomized OR control group OR placebo):ti,ab,kw (Word variations have been searched)

Chinese Database

China National Knowledge Infrastructure（CNKI）

1#: 老年人 + 老年群体 + 中老年人 + 中年人 +高龄
2#: 八段锦 + 八段锦气功 + 气功 + 中国传统运动
3#: 平衡能力 + 身体功能 + 运动功能 + 姿态控制 + 姿态平衡 + 跌倒 + 滑倒
4#: 随机对照试验 + 随机对照研究 + 随机对照 + 对照组 + 控制组
5#: 1# * 2# * 3# * 4#

Chinese biomedical literature database（SinoMed）

("老年人"[全部字段:智能] OR "老年群体"[全部字段:智能] OR "中老年人"[全部字段:智能] OR "中年人"[全部字段:智能] OR "高龄"[全部字段:智能]) AND("八段锦"[全部字段:智能] OR "八段锦气功"[全部字段:智能] OR "气功"[全部字段:智能] OR "中国传统运动"[全部字段:智能]) AND("平衡能力"[全部字段:智能] OR "身体功能"[全部字段:智能] OR "运动功能"[全部字段:智能] OR "姿态控制"[全部字段:智能] OR "姿态平衡"[全部字段:智能] OR "跌倒"[全部字段:智能] OR "滑倒"[全部字段:智能]) AND("随机对照试验"[常用字段:智能] OR "随机对照研究"[常用字段:智能] OR "随机对照"[常用字段:智能] OR "对照组"[常用字段:智能] OR "控制组"[常用字段:智能])

Wanfang med online

((((老年人 OR 老年群体 OR 中老年人 OR 中年人 OR 高龄)) AND (八段锦 OR 八段锦气功 OR 气功 OR 中国传统运动)) AND (平衡能力 OR 身体功能 OR 运动功能 OR 姿态控制 OR 姿态平衡 OR 跌倒 OR 滑倒)) AND 主题=(随机对照试验 OR 随机对照研究 OR 随机对照 OR 对照组 OR 控制组)

B: Characteristics of included studies

Author,year	Types/Design	Research area	Subject characteristics				EG/CG	Intervention programs	Outcome measure	Results
			Numbers	Aged	Genders(M:FM)	Health status				
**Dai et al.,2023[37]**	Article/RCT	China,Community	EG:20<br>CG:20	EG:60.5±3.9<br>CG:60.2±3.2<br>All:60.35±3.57	0:1	GH	EG:Baduanjin<br>CG:NI	12-week intervention, 45-60 minutes 3-5 times per week	**SBT**: Maximum displacement of the center of pressure in the anterior-posterior (AP) direction and in the mediallateral (ML) direction, as well as average velocity<br>**DBT:** Maximum rotation speed, rotation speed, Balance Score, Balance Rating	**SBT**:Sd(P＜0.05)*<br>**DBT**:Sd(P＜0.05)*
**Duan et al.,2024[38]**	Article/RCT	China,Multi-Center	EG:523<br>CG:505	EG:65.36±4.62<br>CG:70.44±5.68<br>All:69.87±5.67	0.29:0.71	GH	EG:BDJ+Fall prevention education<br>CG:Fall prevention education	21-week intervention, 60 minutes 4 times per week	**SBT:** OLST,TST<br>**DBT:** MTEC<br>**PBT:** TUG<br>**BTB:** BBS	**SBT**:Sd(P＜0.05)*<br>**DBT**:Sd(P＜0.05)*<br>**PBT**:Sd(P<0.05)*<br>**BTB**:Sd(P<0.05)*
**ER,YL.2017[39]**	Thesis/RCT	China,Community	EG:66<br>CG:73	EG:67.23±4.54<br>CG:67.22±4.72<br>All:67.22±4.64	0:1	GH	EG:BDJ+Fall prevention education<br>CG:Fall prevention education	12-week intervention, 60 minutes 5 times per week	**SBT:** OLST,TST<br>**DBT:** MTEC<br>**BTB:** BBS	**SBT**:Sd(P>0.05)**<br>**DBT**:Sd(P<0.05)*<br>**BTB**:Sd(P<0.05)*
**Gao,ZP,2020[40]**	Thesis/RCT	China,Community	EG:34<br>CG:34	EG:79.79±4. 18<br>CG:78.88±4.66<br>All:79.34±4.45	0.5:0.5	IH	EG:BDJ<br>CG:HD	12-week intervention, 30 minutes 5 times per week	**SBT**: OLST<br>**DBT**: FTSST<br>**PBT**: TUG,FSST<br>**Fall:** FOF	**SBT**:Sd(P＜0.05)*<br>**DBT**:Sd(P＜0.05)*<br>**PB**:Sd(P＜0.05)*<br>**Fall**:Sd(P＜0.05)*

**Guan,F.2023[41]**	Article/RCT	China,Hospitals	EG:50<br>CG:50	EG:79. 19±6.52<br>CG:70.07±6.45<br>All:74.63±7.93	0.57:0.43	IH	EG: BDJ+Routine rehabilitation or treatment<br>CG:Routine rehabilitation or treatment	12-week intervention, 15 minutes 7 times per week	**BTB:** BBS	**BTB**:Sd(P<0.05)*
**Lui,ST.2015[42]**	Thesis/RCT	China,Hospitals	EG:7<br>CG:8	EG:82. 14±1.68<br>CG:84. 15±2.95<br>All:83.21±2.64	NR	GH	EG:BDJ<br>CG:NI	12-week intervention, 60 minutes 10 times per week	**SBT**:PST<br>**PBT**:TUG	**SBT**:<br>Trajectory length, peripheral area, left and right maximal swing: Sd(P>0.05)**<br>Maximum front/rear swing: Sd(P<0.05)*<br>**PB**:Sd(P<0.05)*
**Wu et al.,2017[43]**	Article/RCT	China,Community	EG:60<br>CG:60	EG:70.63±4.52<br>CG:70.55±4.26<br>All:70.59±4.39	0.3:0.7	IH	EG:BDJ<br>CG:NI	4-week intervention, 60 minutes 14 times per week	**PBT**:TUG<br>**BTB:** BBS<br>**Fall**:Morse	**BTB**:Sd(P＜0.05)*<br>**PB**:Sd(P＜0.05)*<br>**Fall**:Sd(P＜0.05)*
**Xie,DL.2023[44]**	Thesis/RCT	China,Community	EG:83<br>CG:83	EG:69.663±3.369<br>CG:69.386±3.474<br>All:69.52±1.42	0.43:0.57	IH	EG:BDJ<br>CG:NI	52-week intervention, 60 minutes 3 times per week	**BTB**: SPPB	**BTB**:Sd(P＜0.05)*
**Yao,Y.2021 [45]**	Article/RCT	China,Community	EG:50<br>CG:50	EG:71.37±4.90<br>CG:72.58±4.83<br>All:71.975±4.90	0.6:0.4	NR	EG:BDJ<br>CG:NI	NR, 30 minutes 3 times per week	**PBT:** TUG,Balance beam walking,Enhanced Romberg Test<br>**SBT**:OLST<br>**DBT**:MTEC	**SBT**:Sd(P＜0.05)*<br>**DBT**:Sd(P＜0.05)*<br>**PB**:Sd(＜0.05)*

**Yu et al.,2020[46]**	Article/RCT	China,Community	EG:122<br>CG:152	EG:67.40±4.47<br>CG:68.70±5.04<br>All:68. 12±4.84	0.23:0.77	GH	EG:BDJ<br>CG:NI	26-week intervention, 60 minutes 5 times per week	**Fall**:FES-I	**Fall**:Sd(P＜0.05)*
**Zhang,LF.2012[47]**	Thesis/RCT	China,Community	EG:30<br>CG:30	EG:64.60±2.30<br>CG:65. 10±1.90<br>All:64.85±2. 12	0.5:0.5	GH	EG:BDJ<br>CG:NI	26-week intervention, 60 minutes 4 times per week	**SBT**:Postural Equa	**Fall**:Sd(P＜0.06)*
**Zhang LL and Huang CX.,2021[48]**	Article/RCT	China,Hospitals	EG:41<br>CG:41	EG:71.29±4.51<br>CG:70.45±4.29<br>All:70.87±4.42	0.57:0.73	IH	EG: BDJ+Routine rehabilitation or treatment<br>CG:Routine rehabilitation or treatment	8-week intervention, 60 minutes 5 times per week	**DBT**:6WMT	**DBT**:Sd(P＜0.05)*
**Zhao,L.2021[49]**	Thesis/RCT	China,Community	EG:17<br>CG:17	EG:65.82±3.88<br>CG:64.35±3.62<br>All:65.08±3.82	NR	GH	EG:BDJ<br>CG:NI	12-week intervention, 60 minutes 3 times per week	**SBT**:OLST<br>**PBT**: TUG<br>**DBT:**FTSST,MTEC<br>**Fall**:Mobility, Balance, and Falls Risk Assessment Comprehensive Test	**SBT**:Sd(P＜0.05)*<br>**DBT**:Sd(P＜0.05)*<br>**PB**:Sd(＜0.05)*<br>**Fall**:Sd(P＜0.05)*
**Zheng et al.,2024[50]**	Article/RCT	China,Hospitals	EG:30<br>CG:31	EG:64.97±6.67<br>CG:64.39±6.63<br>All:64.68±6.66	0.23:0.77	IH	EG: BDJ+Routine rehabilitation or treatment+HD<br>CG:Routine rehabilitation or treatment+HD	12-week intervention, 30 minutes 5 times per week	**SBT**:OLST<br>**DBT**:FTSST<br>**PBT**: TUG<br>**Fall**:MFES	**SBT**:Sd(P＜0.05)*<br>**DBT**:Sd(P＜0.05)*<br>**PB**:Sd(＜0.05)*<br>**Fall**:Sd(P＜0.05)*

**Zhou et al.,2021[51]**	Article/RCT	China,Hospitals	EG:35<br>CG:35	EG:69. 1±8.5<br>CG:69.5±8.3<br>All:69.3±8.40	0.6:0.4	IH	EG: BDJ+Routine rehabilitation or treatment<br>CG:Routine rehabilitation or treatment	12-week intervention, 60 minutes 5 times per week	**BTB**:BBS	**BTB**:Sd(P＜0.05)*
**Zhou et al.,2020[52]**	Article/RCT	China,Community	EG:30<br>CG:31	EG:59.70±6.23<br>CG:61. 13±6.04<br>All:60.43±6. 18	0.41:0.59	GH	EG:BDJ<br>CG:Walking	12-week intervention, 30 minutes 5 times per week	**PBT**: TUG<br>**SBT**:OLST<br>**BTB**:Tinetti Balance and Gait Assessment Scale	**PB**:Sd(＜0.05)*<br>**SBT**:Sd(＜0.05)*<br>**BTB**:Sd(P＜0.05)*
**Hua,Q.2021[53]**	Article/RCT	China,Hospitals	EG:40<br>CG:40	EG:66.64±10.68<br>CG:65.69±9. 14<br>All:66. 16±9.95	0.5625:0.4375	IH	EG: BDJ+Routine rehabilitation or treatment<br>CG:Routine rehabilitation or treatment	12-week intervention, 40 minutes 3 times per week	**PBT**: TUG<br>**DBT**:6WMT	**PB**:Sd(＜0.05)*<br>**DBT**:Sd(P＜0.05)*
**Li et al.,2019[54]**	Article/RCT	China,Hospitals	EG:44<br>CG:44	EG:65. 1±5. 1<br>CG:65.5±5. 1<br>All:65.3±5. 10	0.32:0.67	IH	EG: BDJ+Routine rehabilitation or treatment<br>CG:Routine rehabilitation or treatment	26-week intervention, 35 minutes 7 times per week	**PBT**: TUG<br>**SBT**:OLST<br>**BTB**:BBS<br>**Fall**:Morse	**PBT**:Sd(＜0.05)*<br>**SBT**:Sd(P＜0.05)*<br>**BTB**:Sd(P＜0.05)*<br>**Fall**:Sd(P＜0.05)*
**Li,QS.2018[55]**	Thesis/RCT	China,Community	EG:10<br>CG:10	EG:61.40±1.84<br>CG:62. 10±2.08<br>All:61.75 ±1.99	NR	GH	EG:BDJ<br>CG:NI	16-week intervention, 60 minutes 5 times per week	**BTB**:Community Mobility-Parkinson's Disease Progression Markers Test<br>**SBT:**Velocity and velocity moment between two-legged and one-legged stance	**BTB**:Sd(P＜0.01)*<br>**SBT**:Sd(P＜0.05)*

**Liao,Y.2018[56]**	Thesis/RCT	China,Community	EG:61<br>CG:59	EG:69.67±5. 15<br>CG:70.03±4.79<br>All:69.85±4.98	0：1	GH	EG:BDJ+HD<br>CG:HD	26-week intervention, 60 minutes 5 times per week	**PBT**: TUG<br>**SBT:**OLST, TST<br>**BTB:**BBS	**PBT**:Sd(＜0.05)*<br>**SBT**:Sd(P＜0.05)*<br>**BTB**:Sd(P＜0.05)*
**Chen et al., 2016[57]**	Article/RCT	China,Hospitals	EG:50<br>CG:50	EG:68. 1±6. 1<br>CG:67.2±5.2<br>All:67.65±5.69	0.5:0.5	GH	EG:BDJ<br>CG:Walking	24-week intervention, 30 minutes 5 times per week	**Fall**:FROP-Com	**Fall**:Sd（P＜0. 05）∗
**Zhou,JT.2022[58]**	Thesis/RCT	China,Community	EG:9<br>CG:13	EG:82.22±3.59<br>CG:80.85±3.63<br>All:81.41±3.68	0:1	IH	EG:BDJ<br>CG:HD	12-week intervention, 60 minutes 3 times per week	**PBT**: TUG<br>**DBT:**6MWT<br>**BTB:** SPPB	**PB**:Sd(＜0.05)*<br>**DBT**:Sd(P＜0.05)*<br>**BTB**:Sd(P＜0.05)*
**Zhou S et al.,2020[59]**	Article/RCT	China,Community	EG:20<br>CG:20	EG:72.67±9.56<br>CG:73.25±8.54<br>All:72.96±9.07	0.425:0.575	IH	EG:BDJ<br>CG:HD	8-week intervention, 40 minutes 5 times per week	**PBT**: TUG<br>**BTB**:BBS<br>**Fall:** Falls Risk Index	**PB**:Sd(＜0.05)*<br>**BTB:**Sd(P＜0.05)*<br>**Fall**:Sd(P＜0.05)*
**Zhuang et al.,2019[60]**	Article/RCT	China,Hospitals	EG:37<br>CG:35	EG:67.85±3.85<br>CG:69.02±2.41<br>All:68.42±3.28	0.475:0.525	IH	EG: BDJ+Routine rehabilitation or treatment<br>CG:Routine rehabilitation or treatment	12-week intervention, 50 minutes 7 times per week	**BTB**:BBS	**BTB**:Sd(P＜0.05)*

**Kuang,XW.2019[61]**	Article/RCT	Cnina, NR	EG:41<br>CG:41	EG:68.68±3.22<br>CG:70.33±3.34<br>All:69.51±3.38	NR	IH	EG: BDJ+Routine rehabilitation or treatment<br>CG:Routine rehabilitation or treatment	12-week intervention, 60 minutes 14 times per week	**BTB**:BBS	**BTB**:Sd(P＜0.06)*
**He et al.,2011[62]**	Article/RCT	Cnina, NR	EG:40<br>CG:40	EG:62.2±2.1<br>CG:63.4±1.5<br>All:62.8±1.92	0:1	GH	EG:BDJ<br>CG:NI	20-week intervention, 45 minutes 7 times per week	**SBT:**OLST	**SBT**:Sd(P＜0.05)*
**Li,RL.2017 [63]**	Article/RCT	China,Hospitals	EG:30<br>CG:30	EG:69.24±6.56<br>CG:66.56±5.31<br>All:67.9±6.12	0.63:0.37	IH	EG:BDJ+general nursing<br>CG:Walking+general nursing	NR	**DBT**:6WMT	**DBT:**Sd(P＜0.05)*
**Shi,XY.2017[64]**	Article/RCT	China,Hospitals	EG:52<br>CG:52	EG:64.7±6.3<br>CG:65.5±6.5<br>All:65.1±6.41	0.51:0.49	IH	EG:BDJ<br>CG:Walking	12-week intervention, 35 minutes 2 times per week	**PBT**:TUG<br>**BTB**:BBS<br>**SBT**:Triangle stand with eyes closed<br>**Fall**:MFES	**BTB**:Sd(P＜0.06)*<br>**PB**:Sd(＜0.05)*<br>**SBT**:Sd(P＜0.05)*<br>**Fall**:Sd(P＜0.05)*

**Xu et al.,2022[65]**	Article/RCT	China,Hospitals	EG:29<br>CG:27	EG:74.90±8.45<br>CG:76. 11±8.34<br>All:75.48±8.42	0.52:0.48	IH	EG: BDJ+Routine rehabilitation or treatment<br>CG:Routine rehabilitation or treatment	8-week intervention, 30 minutes 5 times per week	**DBT**:6WMT	**DBT**:Sd(P＜0.05)*
**Wang et al.,2019[66]**	Article/RCT	China,Hospitals	EG:42<br>CG:42	EG:66.40±4.90<br>CG:66.60±4.70<br>All:66.5±4.80	0.55:0.45	IH	EG:BDJ<br>CG:Walking	12-week intervention, 30 minutes 10 times per week	**BTB**:BBS<br>**DBT**:MTEC<br>**Fall:**MFES	**BTB**:Sd(P＜0.06)*<br>**DBT**:Sd(P＜0.05)*<br>**Fall**:Sd(P＜0.05)*
**Carcelén-Fraile, M. del C. et al., 2021[67]**	Article/RCT	Spain,Community	EG:57<br>CG:60	EG:69.70±6. 15<br>CG:69.75±6.76<br>All:69.73±6.44	NR	GH	EG:BDJ<br>CG:HD	12-week intervention, 60 minutes 2 times per week	**SBT**:mean velocity of the center of pressure displacements,sway area covered by the center of pressure,mediolateral,anteroposterior	**SBT**:<br>SEO: Sd(P>0.05)**<br>XEO: Sd(P>0.05)**<br>SEC: Sd(P>0.05)**<br>YEC: Sd(P>0.05)**<br>YEO: Sd(P＜0.05)*<br>XEC: Sd(P＜0.05)*

**Liu et al., 2016[68]**	Article/QED	China,Community	EG:47<br>CG:48	EG:66.63±5.98<br>CG:67. 1±6. 18<br>All:66.87±6.09	0.79:0.21	GH	EG:BDJ+HD<br>CG:Wlking+HD	12-week intervention, 40 minutes 7 times per week	**PBT**: TUG<br>**SBT**:OLST<br>**BTB**:BBS<br>**Fall**:MFES	**PB**:Sd(＜0.05)*<br>**SBT**:Sd(P＜0.05)*<br>**BTB**:Sd(P＜0.06)*<br>**Fall**:Sd(P＜0.05)*
**Xiao et al., 2017[69]**	Article/RCT	China,Community	EG:47<br>CG:47	All:66.6±7.5	0.43:0.57	GH	EG:BDJ<br>CG:NI	26-week intervention, 60 minutes 7 times per week	**DBT:** 6MWT,30CST<br>**PBT:** TUG<br>**BTB**:BBS	**PB**:Sd(＜0.05)*<br>**SBT**:Sd(P＜0.05)*<br>**BTB**:Sd(P＜0.06)*
**Xiao et al., 2015[70]**	Article/RCT	China,Hospitals	EG:45<br>CG:44	EG:68. 17±2.27<br>CG:66.52±2. 13<br>All:67.35±2.35	0.73:0.27	IH	EG:BDJ<br>CG:Walking	26-week intervention, 45 minutes 4 times per week	**PBT**: TUG<br>**SBT**:6MWT<br>**BTB**:BBS	**PB**:Sd(＜0.05)*<br>**SBT**:Sd(P＜0.05)*<br>**BTB**:Sd(P＜0.05)*

**Ye et al., 2020[71]**	Article/RCT	China,Hospitals	EG:28<br>CG:28	EG:65. 11±6.57<br>CG:63.61±2.63<br>All:64.36±5.06	0.34:0.66	IH	EG: BDJ+Routine rehabilitation or treatment<br>CG:Routine rehabilitation or treatment	12-week intervention, 40 minutes 3 times per week	**SBT**:Sway area covered by the center of pressure,Center of pressure swing perimeter	SBT：<br>OP perimeter:Sd(P>0.05)**<br>OP ellipse area:Sd(P<0.05)*<br>CP perimeter:Sd(P>0.05)**<br>CP ellipsearea:Sd(P>0.05)**
**Yuen et al., 2021[72]**	Article/RCT	China,Hospitals+Home	EG:29<br>CG:29	EG:63. 1±10.6<br>CG:62.0±13. 1<br>All:62.5±11.8	0.5:0.5	IH	EG: BDJ<br>CG:Routine rehabilitation or treatment	16-week intervention, 50 minutes 3 times per week	**PBT**: TUG<br>**DBT**:FTSST<br>**SBT**:Mini-BESTest<br>**Fall**: FES-I	PB: Sd(P＜0.05)*<br>SBT: Sd(P＜0.05)*<br>DBT: Sd(P＜0.05)*<br>Fall: Sd(P>0.05)**
**Ye et al., 2022[73]**	Article/RCT	China,Hospitals	EG:24<br>CG:24	EG:61.63±9.21<br>CG:62.75±6.41<br>All:62. 19±7.87	0.85:0. 15	IH	EG:BDJ+HD<br>CG:HD	24-week intervention, 40 minutes 3 times per week	**BTB**:BBS,Fugl-Meyer	**BTB**:Sd(P＜0.05)*
**Ye et al.,2019[74]**	Article/RCT	China,Hospitals	EG:25<br>CG:25	EG:64.48±7.81<br>CG:63.08±3.65<br>All:63.78±6. 14	0.4:0.6	IH	EG:BDJ<br>CG:NI	12-week intervention, 40 minutes 3 times per week	**SBT**:Degree of standing body sway	**SBT** :<br>CPAP:Sd(P＜0.05)*<br>CPML:Sd(P＞0.05)**<br>OPAP:Sd(P＞0.05)**<br>OPML:Sd(P＞0.05)**

**Tou et al.,2024[75]**	Article/RCT	Singapore,Community	EG:29<br>CG:27	EG:72.9±8.0<br>CG:72.6±5.7<br>All:72.76±6.46	0.05:0.95	IH	EG:BDJ<br>CG:HD	16-week intervention, 60 minutes 3 times per week	**PBT**:TUG<br>**DBT**:30CST<br>**Fall**:Fal lefficacy	**PBT**:Sd(P＞0.05)**<br>**DBT**:Sd(P＞0.05)**<br>**Fall**:Sd(P＞0.05)**
**Jiao,MJ.2024[76]**	Thesis/RCT	China,NR	EG:26<br>CG:26	EG:69±3.92<br>CG:70±5.24<br>All:69.5±4.65	0.38:0.62	GH	EG:BDJ<br>CG:NI	8-week intervention, 60 minutes 2 times per week	**DBT**:MTEC<br>**PBT**:TUG<br>**BTB**:BBS	**DBT**:Sd(P＜0.01)*<br>**PBT**:Sd(P＜0.05)*<br>**BTB**:Sd(P＜0.05)*

Generally health(GH): basic physical and mental health, no cognitive and psychiatric disorders, basic normal balance function, no serious acute illnesses; Imperfect health(IH): elderly people with mental or cognitive disorders, with certain diseases that affect balance disorders, in a period of frailty; EG:experimental group; CG:control group; M:male; FM:female; NI:non-intervention; HD:health education; MTEC:mark time with eyes closed; OLST:One leg stand test with eyes closed or open; TST:Tandem stance test with eyes closed or open; PST:Parallel Stance test with eyes closed or open; TUG:Timed up and go test; BBS:Berg balance scale; FTSST:Five times sit to stand test; FSST:Four square step test; FOF:Fear of falling scale; Morse:Morse fall scale; SPPB:Short physical performance battery; FES-I:Falls efficacy scale-international; 6WMT:6-Minute walk distance test; MFES:Modified Falls Efficacy Scale; FROP-Com:Modifications falls risk for older people in the community; 30CST:30 Seconds sit to stand test; Sd:Mean and standard deviation; *:Significantly different from the control group; **:No significant difference compared to control group;

C: Results of the quality assessment

Author,year	*Item 1*	*Item 2*	*Item 3*	*Item 4*	*Item 5*	*Item 6*	*Item 7*	*Item 8*	*Item 9*	*Item 10*	*Item 11*
**Dai et al.,2023[37]**	Y	Y	N	N	N	N	N	N	Y	Y	Y
**Duan et al.,2024[38]**	Y	Y	N	Y	N	N	N	Y	Y	Y	Y
**ER,YL.2017[39]**	Y	Y	N	Y	N	N	N	Y	Y	Y	Y
**Gao,ZP,2020[40]**	Y	Y	N	Y	N	N	N	Y	Y	Y	Y
**Guan,F.2023[41]**	Y	Y	N	Y	N	N	N	N	Y	Y	Y
**Lui,ST.2015[42]**	Y	Y	N	N	N	N	N	Y	Y	Y	Y
**Wu et al.,2017[43]**	Y	Y	N	Y	N	N	N	Y	N	Y	Y
**Xie,DL.2023[44]**	Y	Y	N	Y	N	N	N	Y	Y	Y	Y
**Yao,Y.2021[45]**	N	Y	N	N	N	N	N	N	Y	Y	Y
**Yu et al.,2020[46]**	Y	N	N	Y	N	N	N	Y	Y	Y	Y
**Zhang,LF.2012[47]**	Y	N	N	Y	N	N	N	Y	Y	Y	Y
**Zhang & Huang ,2021[48]**	Y	Y	N	Y	N	N	N	Y	Y	Y	Y
**Zhao,L.2021[49]**	Y	Y	N	Y	N	N	N	Y	N	Y	Y
**Zheng et al.,2024[50]**	Y	Y	N	N	N	N	N	Y	N	Y	Y
**Zhou et al.,2021[51]**	Y	Y	N	Y	N	N	N	Y	Y	Y	Y
**Zhou et al.,2020[52]**	Y	Y	N	Y	N	N	N	Y	Y	Y	Y
**Hua,Q.2021[53]**	Y	Y	N	Y	N	N	N	Y	Y	Y	Y
**Li et al.,2019[54]**	Y	Y	N	Y	N	N	N	Y	N	Y	Y
**Li,QS.2018[55]**	Y	Y	N	Y	N	N	N	Y	Y	Y	Y
**Liao,Y.2018[56]**	Y	Y	N	Y	N	N	N	Y	Y	Y	Y
**Chen et al., 2016[57]**	Y	Y	N	Y	N	N	N	Y	Y	Y	Y
**Zhou,JT.2022[58]**	Y	Y	Y	Y	Y	N	N	Y	Y	Y	Y
**Zhou S et al.,2020[59]**	Y	Y	N	Y	N	N	N	Y	Y	Y	Y
**Zhuang et al.,2019[60]**	Y	N	N	Y	N	N	N	Y	N	Y	Y
**Kuang,XW.2019[61]**	Y	Y	N	Y	N	N	N	Y	N	Y	Y
**He et al.,2011[62]**	Y	Y	N	N	N	N	N	N	N	Y	Y
**Li,RL.2017[63]**	Y	Y	N	N	N	N	N	Y	Y	Y	Y
**Shi,XY.2017[64]**	N	Y	N	Y	N	N	N	Y	Y	Y	Y

**Xu et al.,2022[65]**	*Y*	*Y*	*N*	*Y*	*N*	*N*	*N*	*Y*	*N*	*Y*	*Y*
**Wang et al.,2019[66]**	*Y*	*Y*	*N*	*Y*	*N*	*N*	*N*	*Y*	*N*	*Y*	*Y*
**Carcelén-Fraile, M. del C. et al., 2021[67]**	*Y*	*Y*	*Y*	*Y*	*N*	*N*	*Y*	*Y*	*Y*	*Y*	*Y*
**Liu et al., 2016[68]**	*NA*	*NA*	*NA*	*NA*	*NA*	*NA*	*NA*	*NA*	*NA*	*NA*	*NA*
**Xiao et al., 2017[69]**	*Y*	*Y*	*N*	*Y*	*N*	*N*	*N*	*Y*	*N*	*Y*	*Y*
**Xiao et al., 2015[70]**	*Y*	*Y*	*N*	*Y*	*N*	*N*	*Y*	*Y*	*Y*	*Y*	*Y*
**Ye et al., 2020[71]**	*Y*	*Y*	*N*	*N*	*N*	*N*	*N*	*Y*	*Y*	*Y*	*Y*
**Yuen et al., 2021[72]**	*Y*	*Y*	*N*	*Y*	*N*	*N*	*Y*	*Y*	*Y*	*Y*	*Y*
**Ye et al., 2022[73]**	*Y*	*Y*	*Y*	*Y*	*N*	*N*	*Y*	*Y*	*Y*	*Y*	*Y*
**Ye et al.,2019[74]**	*Y*	*Y*	*N*	*Y*	*N*	*N*	*Y*	*N*	*Y*	*Y*	*Y*
**Tou et al.,2024[75]**	*Y*	*Y*	*Y*	*Y*	*N*	*N*	*Y*	*Y*	*Y*	*Y*	*Y*
**Jiao,MJ.2024[76]**	*Y*	*Y*	*N*	*Y*	*N*	*N*	*N*	*Y*	*N*	*Y*	*Y*

Item 1: eligibility criteria were specified; Item 2:subjects were randomly allocated to groups (in a crossover study, subjects were randomly allocated an order in which treatments were received) ; Item 3:allocation was concealed; Item 4:the groups were similar at baseline regarding the most important prognostic indicators; Item 5:there was blinding of all subjects; Item 6:there was blinding of all therapists who administered the therapy ; Item 7: there was blinding of all assessors who measured at least one key outcome; Item 8: measures of at least one key outcome were obtained from more than 85% of the subjects initially allocated to groups; Item 9: all subjects for whom outcome measures were available received the treatment or control condition as allocated or, where this was not the case, data for at least one key outcome was analysed by “intention to treat”; Item 10:the results of between-group statistical comparisons are reported for at least one key outcome; Item 11:the study provides both point measures and measures of variability for at least one key outcome; NA:Not applicable

D: Control group subgroup results

Study or Subgroup	Experimental Mean	Experimental SD	Experimental Total	Control Mean	Control SD	Control Total	Weight	Std. Mean Difference IV, Random, 95% CI
**3.1.1 Health education**								
Duan et al.,2024	53.01	3.17	523	47.92	3.4	505	6.1%	1.55 [1.41, 1.69]
Er,YL.2017	50.11	4.07	66	47	5.56	73	5.5%	0.63 [0.29, 0.97]
Ye et al., 2022a	44.23	5.34	20	40.15	4.38	20	4.3%	0.82 [0.17, 1.47]
Ye et al., 2022b	45.89	4.73	24	42.21	4.73	24	4.5%	0.77 [0.18, 1.35]
Zhou et al.,2020	73.64	9.83	24	66.02	10.95	24	4.5%	0.72 [0.13, 1.31]
Zhou S et al., 2020	9	1.41	9	7.23	2.98	13	3.4%	0.69 [-0.19, 1.57]
Zhou,JT.2022	25.83	1.32	30	23.26	1.34	31	4.4%	1.91 [1.30, 2.52]
**Subtotal (95% CI)**			**696**			**690**	**32.8%**	**1.03 [0.60, 1.47]**
Heterogeneity: Tau^2 = 0.27; Chi^2 = 41.06, df = 6 (P < 0.00001); I^2 = 85%								
Test for overall effect: Z = 4.64 (P < 0.00001)								
**3.1.2 Routine care or rehabilitation**								
Guan,F.2023	53.28	5.61	50	47.46	6.29	50	5.3%	0.97 [0.55, 1.38]
Kuang, XW.2019	49.81	6.52	41	41.22	5.37	41	5.0%	1.42 [0.94, 1.91]
Yuen et al., 2021	22	3.7	29	18.5	5	29	4.7%	0.79 [0.25, 1.32]
Zhou et al.,2021	81.68	13.62	35	72.31	12.73	35	5.0%	0.70 [0.22, 1.19]
Zhuang et al.,2019	40.53	10.26	37	29.57	9.68	35	4.9%	1.09 [0.59, 1.58]
**Subtotal (95% CI)**			**192**			**190**	**24.8%**	**1.00 [0.75, 1.24]**
Heterogeneity: Tau^2 = 0.02; Chi^2 = 5.12, df = 4 (P = 0.27); I^2 = 22%								
Test for overall effect: Z = 8.04 (P < 0.00001)								
**3.1.3 Walking**								
Liu et al., 2016	53.57	2.676	47	51.81	4.144	48	5.3%	0.50 [0.09, 0.91]
Shi,XY.2017	53.5	12	52	48.8	11	52	5.4%	0.41 [0.02, 0.79]
Wang et al.,2019	48.18	3.22	42	45.81	4.14	42	5.2%	0.63 [0.19, 1.07]
Xiao et al.,2015	50.3	0.85	45	47	2.15	44	4.8%	2.01 [1.50, 2.52]
**Subtotal (95% CI)**			**186**			**186**	**20.6%**	**0.87 [0.21, 1.53]**
Heterogeneity: Tau^2 = 0.40; Chi^2 = 27.81, df = 3 (P < 0.00001); I^2 = 89%								
Test for overall effect: Z = 2.59 (P = 0.010)								
**3.1.4 Regular activities**								
Jiao,MJ.2024	53.962	1.708	26	51.269	1.909	26	4.4%	1.46 [0.85, 2.08]
Li,QS.2018	16	0.001	10	14.7	0.34	10	1.2%	5.18 [3.18, 7.17]
Wu et al.,2017	48.02	4.123	60	43.02	3	60	5.3%	1.38 [0.98, 1.78]
Xiao et al.,2017	47.73	5.48	47	41.5	10.63	47	5.2%	0.73 [0.31, 1.15]
Xie,DL.2023	9.48	1.61	83	8.28	1.55	83	5.6%	0.76 [0.44, 1.07]
**Subtotal (95% CI)**			**226**			**226**	**21.7%**	**1.33 [0.75, 1.92]**
Heterogeneity: Tau^2 = 0.33; Chi^2 = 26.32, df = 4 (P < 0.0001); I^2 = 85%								
Test for overall effect: Z = 4.48 (P < 0.00001)								
**Total (95% CI)**			**1300**			**1292**	**100.0%**	**1.04 [0.81, 1.28]**
Heterogeneity: Tau^2 = 0.24; Chi^2 = 126.74, df = 20 (P < 0.00001); I^2 = 84%								
Test for overall effect: Z = 8.60 (P < 0.00001)								
Test for subgroup differences: Chi^2 = 1.33, df = 3 (P = 0.72), I^2 = 0%								

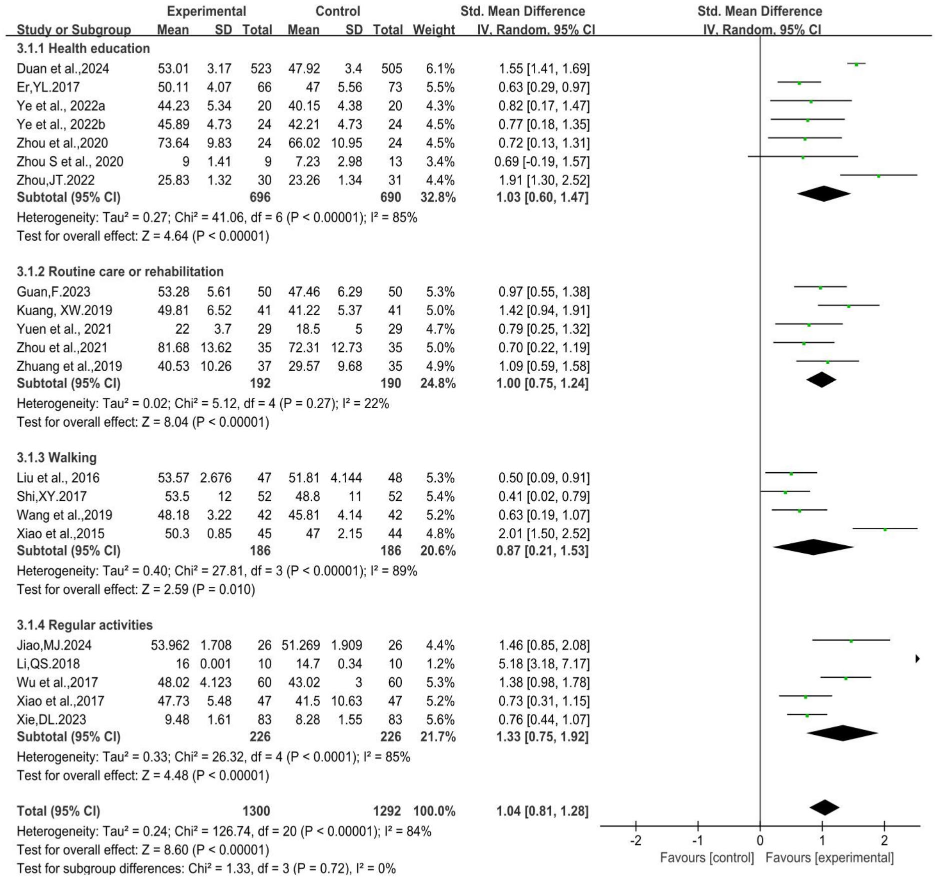

A-BTB

Study or Subgroup	Experimental Mean	Experimental SD	Experimental Total	Control Mean	Control SD	Control Total	Weight	Std. Mean Difference IV, Random, 95% CI
**3.2.1 Health education**								
Duan et al.,2024	10.87	4.43	523	12.88	5.01	505	5.2%	-0.43 [-0.55, -0.30]
Gao,ZP. 2020a	12.16	0.64	34	14.74	0.79	34	4.1%	-3.55 [-4.32, -2.77]
Gao,ZP. 2020b	12.28	0.64	34	15.24	0.69	34	3.8%	-4.40 [-5.29, -3.50]
Tou et al.,2024	10.94	3.28	29	10.58	2.59	27	4.6%	0.12 [-0.41, 0.64]
Zhou S et al., 2020	10.23	2.34	20	13.05	2.38	20	4.3%	-1.17 [-1.85, -0.49]
Zhou,JT.2022	14.23	2.5	9	16.98	5.29	13	3.8%	-0.60 [-1.47, 0.27]
**Subtotal (95% CI)**			**649**			**633**	**25.9%**	**-1.63 [-2.78, -0.49]**
Heterogeneity: Tau^2 = 1.93; Chi^2 = 141.19, df = 5 (P < 0.00001); I^2 = 96%								
Test for overall effect: Z = 2.79 (P = 0.005)								
**3.2.2 Routine care or rehabilitaion**								
Hua et al.,2021	6.79	1.63	40	9.19	1.92	40	4.7%	-1.33 [-1.82, -0.85]
Li et al.,2019	7.84	1.31	44	10.44	1.33	44	4.7%	-1.95 [-2.46, -1.44]
Yuen et al., 2021	15.2	7.7	29	16.8	11	29	4.7%	-0.17 [-0.68, 0.35]
Zheng et al.,2024	9.29	1.73	30	11.61	2.47	31	4.6%	-1.07 [-1.61, -0.53]
**Subtotal (95% CI)**			**143**			**144**	**18.7%**	**-1.13 [-1.86, -0.41]**
Heterogeneity: Tau^2 = 0.48; Chi^2 = 24.06, df = 3 (P < 0.0001); I^2 = 88%								
Test for overall effect: Z = 3.06 (P = 0.002)								
**3.2.3 Walking**								
Liu et al., 2016	7.127	1.261	47	8.625	1.393	48	4.8%	-1.12 [-1.55, -0.68]
Shi,XY.2017	7.08	1.04	52	7.53	1.15	52	4.9%	-0.41 [-0.80, -0.02]
Xiao et al., 2015	11.2	1.7	45	13.1	3.1	44	4.8%	-0.76 [-1.19, -0.32]
Zhou et al.,2020	4.37	1	30	5.19	1.58	31	4.7%	-0.61 [-1.12, -0.10]
**Subtotal (95% CI)**			**174**			**175**	**19.2%**	**-0.72 [-1.03, -0.41]**
Heterogeneity: Tau^2 = 0.05; Chi^2 = 5.91, df = 3 (P = 0.12); I^2 = 49%								
Test for overall effect: Z = 4.57 (P < 0.00001)								
**3.2.4 Regular activities**								
Jiao,MJ.2024	9.276	1.979	26	11.78	1.312	26	4.4%	-1.47 [-2.09, -0.85]
Liu,ST.2023	13.49	0.62	7	15.08	1.37	8	3.2%	-1.37 [-2.54, -0.21]
Wu et al.,2017	12.02	3.921	60	13.25	2.088	60	5.0%	-0.39 [-0.75, -0.03]
Xiao et al.,2017	6.79	2.36	47	7.83	4.52	47	4.9%	-0.29 [-0.69, 0.12]
Yao et al.,2021a	60.16	14.36	50	70.79	14.3	50	4.9%	-0.74 [-1.14, -0.33]
Yao et al.,2021b	87.16	3.01	50	91.09	3.67	50	4.8%	-1.16 [-1.59, -0.74]
Yao et al.,2021c	10.48	2.76	50	7.94	3.21	50	4.9%	0.84 [0.43, 1.25]
Zhao,L.2021	7.57	1.27	17	9.14	1.19	17	4.2%	-1.25 [-1.99, -0.50]
**Subtotal (95% CI)**			**307**			**308**	**36.2%**	**-0.68 [-1.22, -0.13]**
Heterogeneity: Tau^2 = 0.52; Chi^2 = 68.72, df = 7 (P < 0.00001); I^2 = 90%								
Test for overall effect: Z = 2.45 (P = 0.01)								
**Total (95% CI)**			**1273**			**1260**	**100.0%**	**-1.00 [-1.33, -0.67]**
Heterogeneity: Tau^2 = 0.53; Chi^2 = 260.14, df = 21 (P < 0.00001); I^2 = 92%								
Test for overall effect: Z = 5.94 (P < 0.00001)								
Test for subgroup differences: Chi^2 = 3.30, df = 3 (P = 0.35), I^2 = 9.2%								

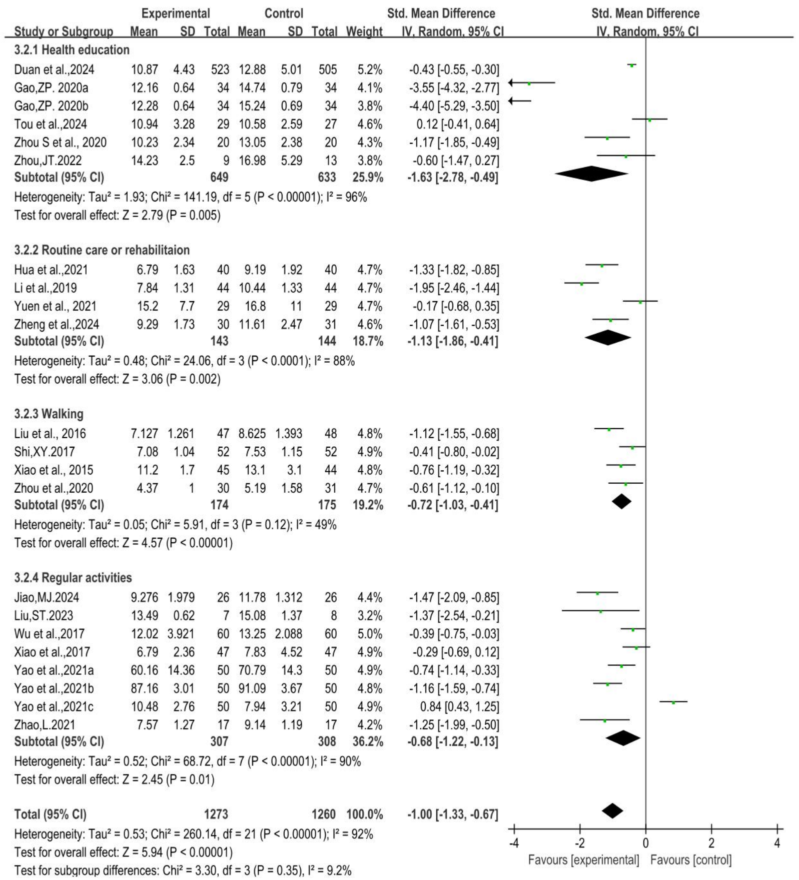

B-PBT

Study or Subgroup	Experimental Mean	Experimental SD	Experimental Total	Control Mean	Control SD	Control Total	Weight	Std. Mean Difference IV, Random, 95% CI
**3.3.1 Health education**								
Duan et al.,2024	4.805	3.19	523	3.02	2.96	505	7.8%	0.58 [0.45, 0.70]
Duan et al.,2024a	11.66	8.45	523	5.9	7.88	505	7.8%	0.70 [0.58, 0.83]
Duan et al.,2024b	15.075	9.476	523	8.87	10.36	505	7.8%	0.63 [0.50, 0.75]
Duan et al.,2024c	33.22	16.1	523	16.11	16.53	505	7.7%	1.05 [0.92, 1.18]
Er,YL.2017	6.005	4.38	66	3.415	2.31	73	6.2%	0.75 [0.40, 1.09]
Er,YL.2017a	10.59	9.3	66	6.54	6.05	73	6.3%	0.52 [0.18, 0.86]
Er,YL.2017b	15.725	10.53	66	11.885	13.87	73	6.3%	0.31 [-0.03, 0.64]
Er,YL.2017c	23	13.18	66	19.81	17.75	73	6.3%	0.20 [-0.13, 0.54]
Gao,ZP.2020	6.61	1.38	34	3.05	1.33	34	3.9%	2.60 [1.94, 3.25]
**Subtotal (95% CI)**			**2390**			**2346**	**60.1%**	**0.73 [0.52, 0.93]**
Heterogeneity: Tau² = 0.08; Chi² = 79.46, df = 8 (P < 0.00001); I² = 90%								
Test for overall effect: Z = 6.85 (P < 0.00001)								
**3.3.2 Routine care or rehabilitaion**								
Li et al.,2019	6.44	1.01	44	3.88	1.26	44	4.7%	2.22 [1.69, 2.76]
Zheng et al.,2024	5.95	1.17	30	4.27	1.24	31	4.5%	1.38 [0.81, 1.94]
**Subtotal (95% CI)**			**74**			**75**	**9.3%**	**1.80 [0.97, 2.63]**
Heterogeneity: Tau² = 0.28; Chi² = 4.57, df = 1 (P = 0.03); I² = 78%								
Test for overall effect: Z = 4.26 (P < 0.0001)								
**3.3.3 Walking**								
Liu et al., 2016	6.17	2.995	47	3.958	1.867	48	5.6%	0.88 [0.46, 1.30]
Shi,XY.2017	6.32	2.23	52	5.48	2.16	52	5.9%	0.38 [-0.01, 0.77]
Zhou,J.2020	10.53	2.4	30	9.1	2.02	31	4.9%	0.64 [0.12, 1.15]
**Subtotal (95% CI)**			**129**			**131**	**16.4%**	**0.62 [0.32, 0.93]**
Heterogeneity: Tau² = 0.02; Chi² = 2.95, df = 2 (P = 0.23); I² = 32%								
Test for overall effect: Z = 3.98 (P < 0.0001)								
**3.3.4 Regular activities**								
He et al.,2011	10.2	3.3	40	7.23	4.3	40	5.3%	0.77 [0.31, 1.22]
Yao et al., 2021	11.08	3.71	50	6.879	2.99	50	5.6%	1.24 [0.81, 1.67]
Zhao,L.2021	6.83	1.43	17	4.65	1.47	17	3.3%	1.47 [0.70, 2.24]
**Subtotal (95% CI)**			**107**			**107**	**14.2%**	**1.10 [0.71, 1.49]**
Heterogeneity: Tau² = 0.05; Chi² = 3.31, df = 2 (P = 0.19); I² = 40%								
Test for overall effect: Z = 5.55 (P < 0.00001)								
**Total (95% CI)**			**2700**			**2659**	**100.0%**	**0.87 [0.69, 1.05]**
Heterogeneity: Tau² = 0.11; Chi² = 127.09, df = 16 (P < 0.00001); I² = 87%								
Test for overall effect: Z = 9.41 (P < 0.00001)								
Test for subgroup differences: Chi² = 9.66, df = 3 (P = 0.02), I² = 68.9%								

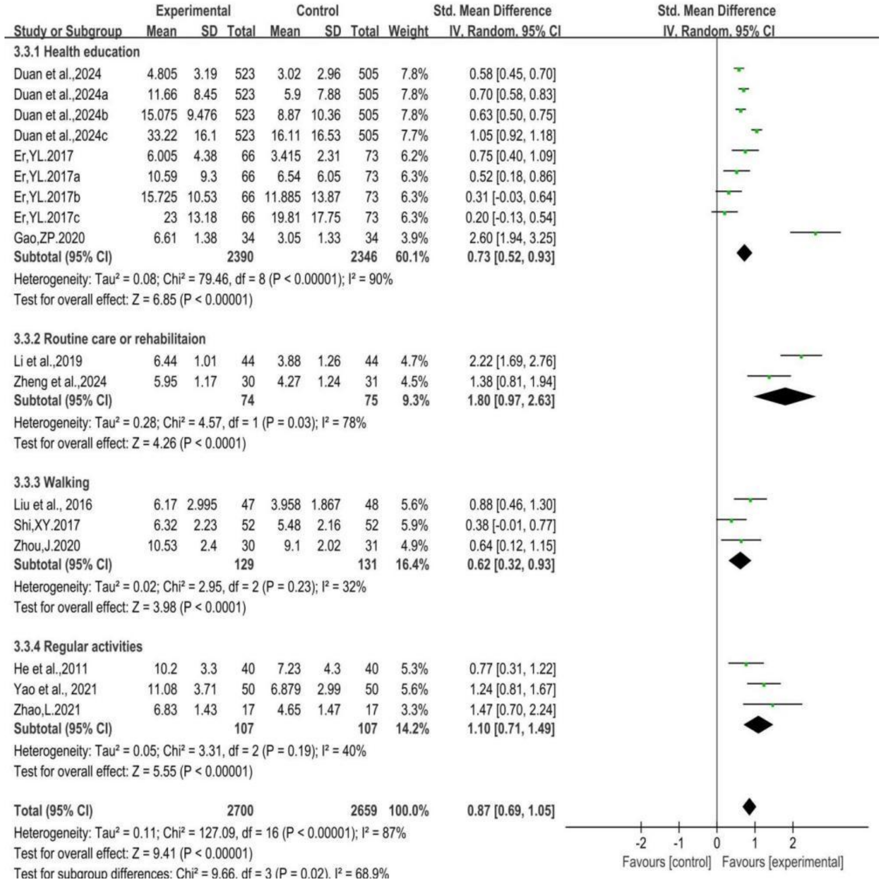

C-SBT

Study or Subgroup	Experimental Mean	Experimental SD	Experimental Total	Control Mean	Control SD	Control Total	Weight	Std. Mean Difference IV, Random, 95% CI
**3.4.1 Health education**								
Duan et al.,2024	7.37	3.97	523	6.14	4.34	505	8.4%	0.30 [0.17, 0.42]
Er,YL.2017	9.75	3.91	66	8.23	4.35	73	7.8%	0.36 [0.03, 0.70]
Tou et al.,2024	14.75	4.47	29	15.6	5.8	27	7.1%	-0.16 [-0.69, 0.36]
**Subtotal (95% CI)**			**618**			**605**	**23.3%**	**0.26 [0.06, 0.46]**
Heterogeneity: Tau² = 0.01; Chi² = 3.03, df = 2 (P = 0.22); I² = 34%								
Test for overall effect: Z = 2.53 (P = 0.01)								
**3.4.2 Routine care or rehabilitaion**								
Hua et al.,2021	330.26	38.34	40	311.62	28.53	40	7.4%	0.55 [0.10, 0.99]
Xu et al.,2022	322.86	106.42	29	330.89	99.97	27	7.1%	-0.08 [-0.60, 0.45]
Zhang et al.,2021	107.84	12.34	41	89.95	9.67	41	7.2%	1.60 [1.10, 2.10]
**Subtotal (95% CI)**			**110**			**108**	**21.6%**	**0.69 [-0.23, 1.61]**
Heterogeneity: Tau² = 0.60; Chi² = 21.28, df = 2 (P < 0.0001); I² = 91%								
Test for overall effect: Z = 1.47 (P = 0.14)								
**3.4.3 Walking**								
Li,RL.2017	550.6	40.51	30	368.71	32.24	30	4.7%	4.90 [3.86, 5.94]
Wang et al.,2019	25.83	3.82	42	21.02	4.34	42	7.3%	1.17 [0.70, 1.63]
Xiao et al., 2015	372.6	19.7	45	365.9	23.5	44	7.5%	0.31 [-0.11, 0.72]
**Subtotal (95% CI)**			**117**			**116**	**19.6%**	**2.05 [0.19, 3.90]**
Heterogeneity: Tau² = 2.57; Chi² = 65.20, df = 2 (P < 0.00001); I² = 97%								
Test for overall effect: Z = 2.16 (P = 0.03)								
**3.4.4 Regular activities**								
Jiao,MJ.2024	8.014	1.608	26	6.608	1.24	26	6.8%	0.96 [0.39, 1.54]
Xiao et al.,2017	14.16	3.27	47	11.45	3.96	47	7.5%	0.74 [0.32, 1.16]
Xiao et al.,2017b	457.38	87.51	47	408.95	79.28	47	7.5%	0.58 [0.16, 0.99]
Yao et al.,2021	13.63	4.05	50	9.1	3.87	50	7.5%	1.13 [0.71, 1.56]
Zhao,L.2021	15.88	2.5	17	12.94	3.1	17	6.2%	1.02 [0.30, 1.74]
**Subtotal (95% CI)**			**187**			**187**	**35.5%**	**0.85 [0.64, 1.07]**
Heterogeneity: Tau² = 0.00; Chi² = 4.07, df = 4 (P = 0.40); I² = 2%								
Test for overall effect: Z = 7.77 (P < 0.00001)								
**Total (95% CI)**			**1032**			**1016**	**100.0%**	**0.85 [0.50, 1.19]**
Heterogeneity: Tau² = 0.36; Chi² = 132.75, df = 13 (P < 0.00001); I² = 90%								
Test for overall effect: Z = 4.85 (P < 0.00001)								
Test for subgroup differences: Chi² = 18.52, df = 3 (P = 0.0003), I² = 83.8%								

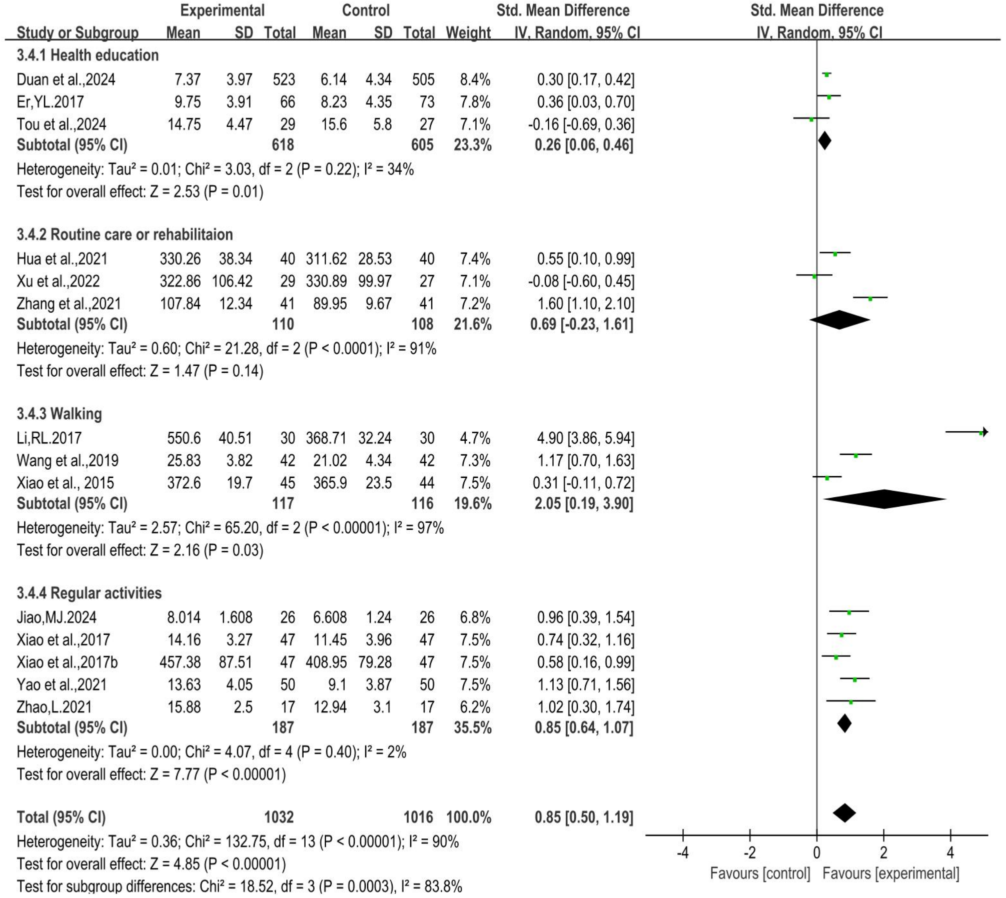

D-DBT

E: Dosage subgroup results

		BTB				PBT				SBT				DBT			
		SMD 95%CI	***Heterogeneity***	***S***	***N***	***SMD 95%CI***	***Heterogeneity***	***S***	***N***	***SMD 95%CI***	***Heterogeneity***	***S***	***N***	***SMD 95%CI***	***Heterogeneity***	***S***	***N***
	ALL	1.04 [0.81, 1.28]	I^2=84%	21	2592	-1.10 [-1.45,-0.76]	I^2=91%	19	2233	0.87 [0.69, 1.05]	I^2=87%	17	5359	0.64 [0.32,0.79]	I^2=80%	11	1832
**TT**	4-8 week	1.52 [1.22,1.81]	I^2=3%	3	233	-0.97 [-1.69,-0.25]	I^2=81%	3	212	NI	I^2=NI	NI	NI	1.30 [0.68,1.92]	I^2=62%	2	134
	12-16 week	0.84 [0.59, 1.10]	I^2=68%	12	886	-1.27 [-1.87,-0.67]	I^2=92%	12	722	0.85 [0.50,1.20]	I^2=85%	10	979	0.57 [0.13,1.00]	I^2=76%	5	393
	20-26 week	1.18 [0.69,1.66]	I^2=86%	5	1307	-0.82 [-1.40,-0.24]	I^2=91%	4	1299	0.89 [0.65, 1.13]	I^2=92%	6	4280	0.42 [0.22,0.63]	I^2=43%	4	1305
	>27 week	0.76 [0.44,1.07]	I^2=NA	1	166	NI	I^2=NI	NI	NI	NI	I^2=NI	NI	NI	NI	I^2=NI	NI	NI
**FPW**	<3 times	1.46 [0.85,2.08]	I^2=NI	1	52	-0.91 [-1.95,0.13]	I^2=88%	2	156	NI	I^2=NI	NI	NI	0.96 [0.39,1.54]	I^2=NI	1	52
	3 times	0.67 [0.48,0.86]	I^2=0%	6	446	-0.52 [-1.11,0.06]	I^2=91%	8	550	0.98 [0.30,1.67]	I^2=82%	3	238	0.63 [0.05, 1.21]	I^2=81%	4	270
	4 times	1.71 [1.28,2. 14]	I^2=65%	2	1117	-0.52 [-0.82,-0.23]	I^2=52%	2	1117	0.74 [0.53,0.94]	I^2=90%	4	4212	0.30 [0.18,0.41]	I^2=0%	2	1117
	5 times	1.38 [0.63,2. 13]	I^2=87%	5	330	-2.13 [-3.44,-0.81]	I^2=95%	5	298	0.86 [0.40,1.32]	I^2=89%	7	746	0.97 [-0.24,2.18]	I^2=94%	2	221
	7 times	0.80 [0.55,1.06]	I^2=27%	5	361	-1.11 [-2.03,-0.18]	I^2=92%	3	277	1.28 [0.43,2. 12]	I^2=90%	3	263	0.81 [0.48,1.15]	I^2=44%	3	272
	≥10times	1.14 [0.64,1.65]	I^2=74%	3	286	-0.72 [-1.63,0.19]	I^2=60%	2	135	NI	I^2=NI	NI	NI	NI	I^2=NI	NI	NI

**DPS**	＜30 min	0.97 [0.55,1.38]	I^2=NA	1	100	NI	I^2=NI	NI	NI	NI	I^2=NI	NI	NI	NI	I^2=NI	NI	NI
	30-39 min	0.51 [0.21,0.80]	I^2=0%	3	188	-1.40 [-2.22,-0.58]	I^2=96%	9	750	1.39 [0.72,2.05]	I^2=91%	6	482	1.15 [0.84,1.46]	I^2=0%	2	184
	40-49 min	1.17 [0.52,1.82]	I^2=87%	5	341	-1.07 [-1.32,-0.81]	I^2=9%	4	304	0.83 [0.52,1.14]	I^2=0%	2	175	0.42 [0.11,0.72]	I^2=0%	2	169
	50-60 min	1.09 [0.80,1.38]	I^2=84%	13	1963	-0.53 [-0.79,-0.26]	I^2=67%	9	1479	0.65 [0.48,0.82]	I^2=84%	9	4702	0.64 [0.32,0.96]	I^2=82%	8	1579
**TPW**	≤150 min	0.95 [0.63,1.26]	I^2=73%	9	687	-1.23 [-1.90,-0.56]	I^2=95%	11	852	1.22 [0.54,1.89]	I^2=89%	5	394	0.90 [0.52,1.24]	I^2=47%	3	232
	151-250 min	1.60 [1.35,1.84]	I^2=20%	2	1089	-0.85 [-1.32,-0.37]	I^2=87%	7	1357	0.96 [0.70,1.21]	I^2=92%	6	4234	0.30 [0.00,0.60]	I^2=56%	4	1207
	251-350 min	0.86 [0.48,1.24]	I^2=74%	7	520	-1.12 [-1.55,-0.68]	I^2=NA	1	95	0.55 [0.33,0.77]	I^2=53%	6	731	1.03 [0.27,1.78]	I^2=89%	3	305
	＞350 min	1.17 [0.73,1.62]	I^2=68%	3	296	-0.43 [-0.78,-0.07]	I^2=33%	3	229	NI	I^2=NI	NI	NI	0.66 [0.36,0.95]	I^2=0%	2	188

TT: Total weeks of intervention; FPW: Frequency of per-week interventions; DPS: Duration of training per time; TPW: Training duration per week; NI: No information; NA: Not applicable; S: Sample size; N: Number of participants

F: Sensitivity analysis results

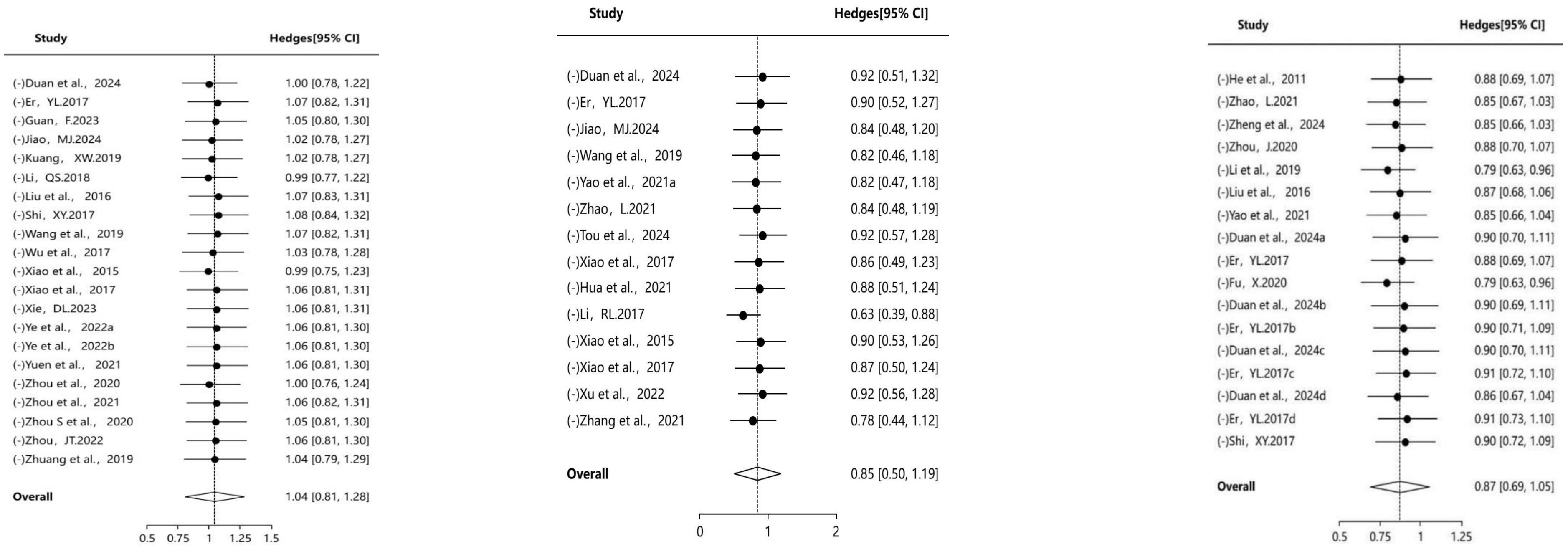

A-BTB **B-DBT** **C-SBT**

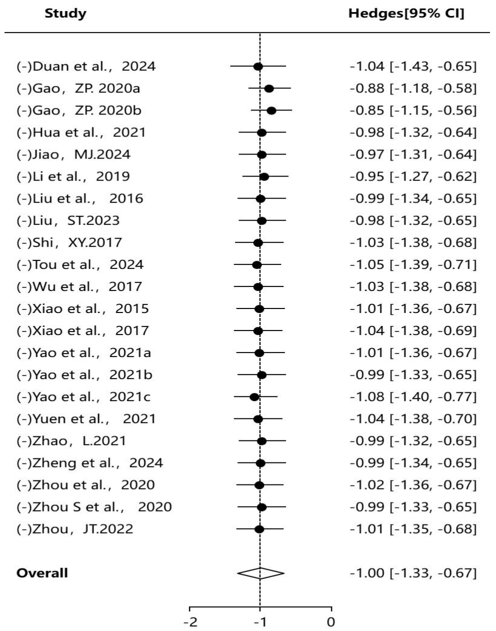

D-PBT

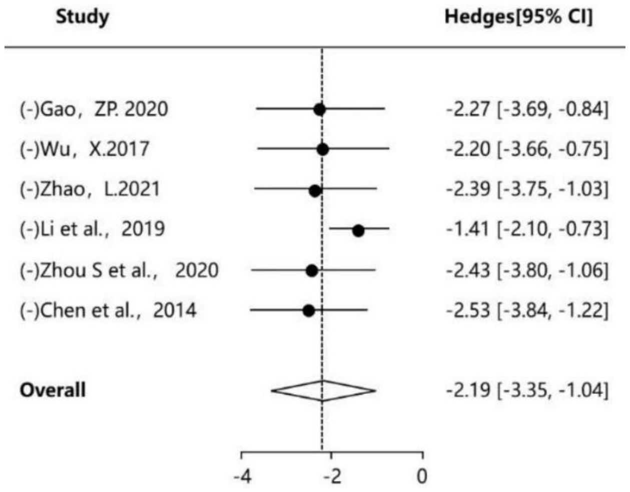

E-Fall risk

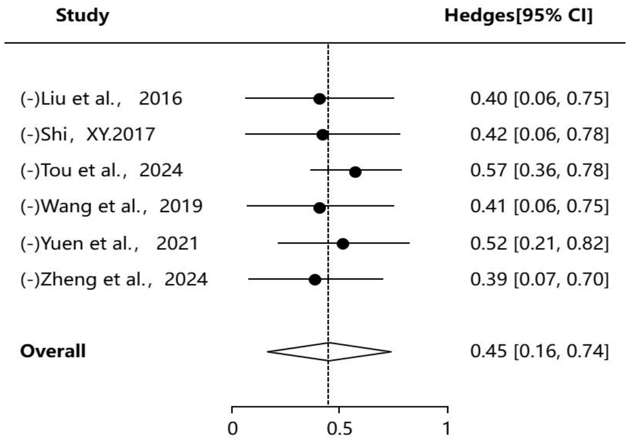

F-Falls effectiveness

G: Funnel plots

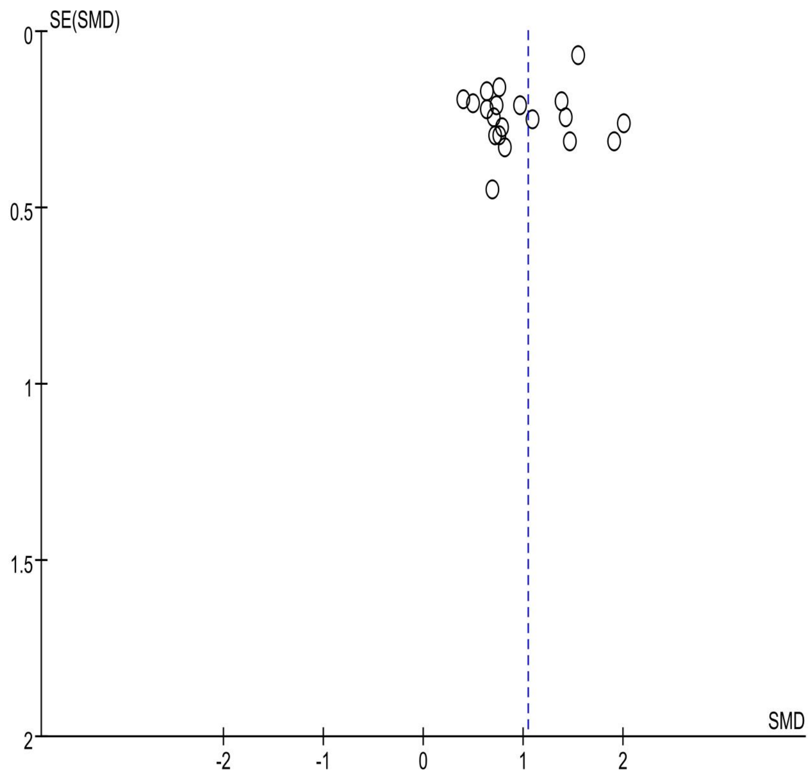

BTB

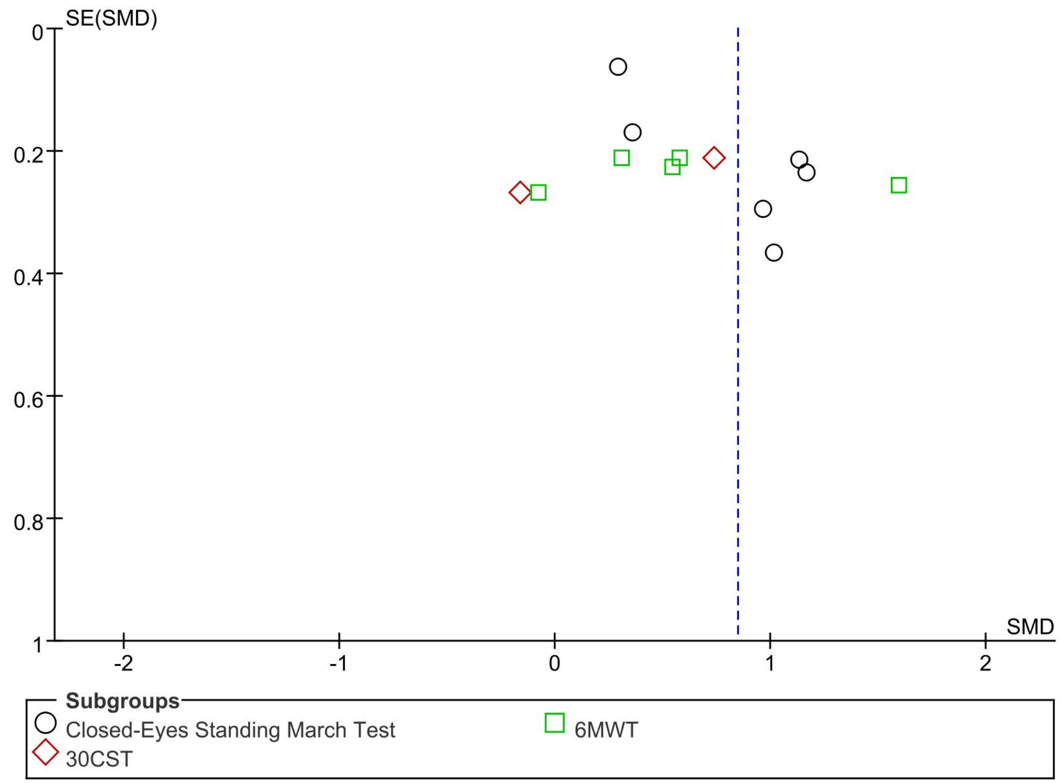

DBT

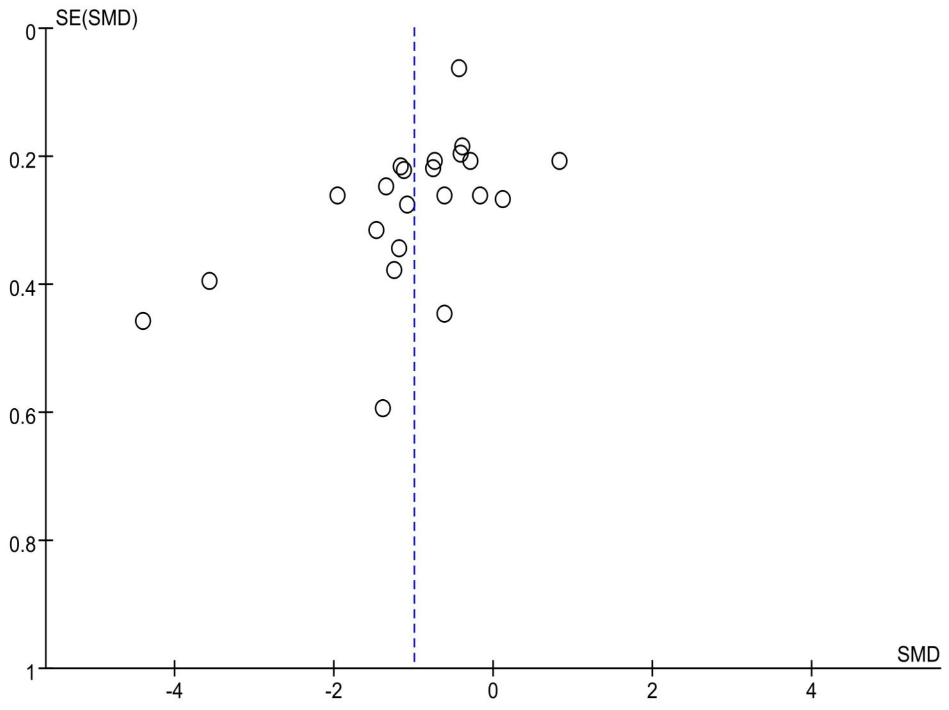
SE(SMD)
0
0.2
0.4
0.6
0.8
1
SMD
-4
-2
0
2
4

PBT

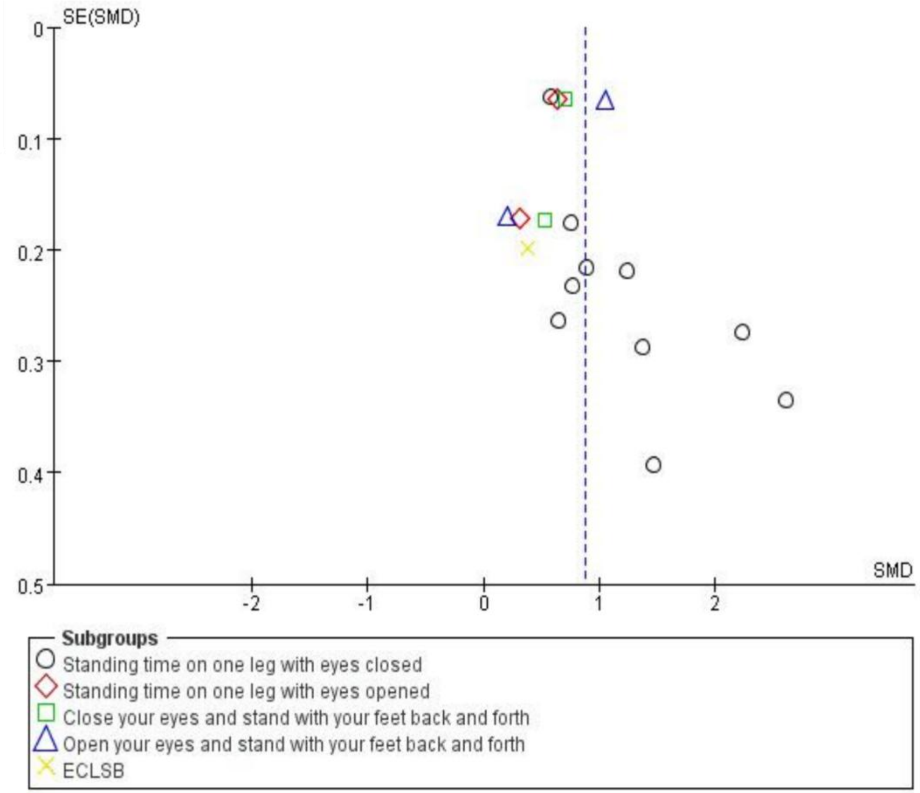
SE(SMD)
0
0.1
0.2
0.3
0.4
0.5
SMD
-2
-1
0
1
2
Subgroups
Standing time on one leg with eyes closed
Standing time on one leg with eyes opened
Close your eyes and stand with your feet back and forth
Open your eyes and stand with your feet back and forth
ECLSB

SBT
